# Data Stream and Hospital-Level Variation in Intensive Care Unit Vital-Sign Record Density Across Medical Information Mart for Intensive Care IV and eICU Collaborative Research Database: Retrospective Data Quality Study

**DOI:** 10.2196/99638

**Published:** 2026-10-29

**Authors:** Yngve Mikkelsen

**Affiliations:** 1 Saïd Business School University of Oxford Oxford, England United Kingdom

**Keywords:** clinical informatics, data quality, electronic health records, informative observation, intensive care, process measures, secondary use

## Abstract

**Background:**

Electronic health record–derived process measures such as vital-sign record density are used for quality monitoring, risk prediction, and cross-system benchmarking, but whether they retain consistent meaning across sites and data streams is rarely tested.

**Objective:**

This study aimed to determine whether vital-sign record density varies primarily by data stream, database, contributing site, or care unit, and what follows for comparative use.

**Methods:**

We retrospectively analyzed the Medical Information Mart for Intensive Care IV (MIMIC-IV) version 3.1 and the eICU Collaborative Research Database (eICU-CRD) version 2.0. Record density and timing metrics were computed for the first 24 intensive care unit hours from nurse-charted streams and, in eICU-CRD, from the monitor stream. Heart rate was primary; oxygen saturation and respiratory rate were replication variables. Variance was decomposed by database, hospital, and unit using η^2^ with hospital-clustered bootstrap inference and mixed-effects sensitivity analyses. A hospital-level plausibility floor of 12 nurse-charted heart rate observations per 24 hours was applied for the principal analysis; unrestricted and alternative-floor analyses are reported as sensitivities.

**Results:**

A pooled-item MIMIC-IV heart rate count included alarm-limit documentation events, so label-based pooling mixes the two. In 117,572 eICU-CRD stays present in both streams, nurse- and monitor-derived counts were almost unrelated (Spearman ρ=0.024), with bottom-decile overlap at chance. Between-database η^2^ for record count was 0.960 against the eICU monitor stream and 0.026 against the nurse stream. Eight of 68 eICU-CRD hospitals fell below the plausibility floor and were excluded from principal estimates, a restriction introduced during review. Among the remaining 60 hospitals (88,560 stays), hospital η^2^ for record count was 0.354 (95% CI 0.259-0.450) against unit-within-hospital η^2^ of 0.043; for the interval-structure metrics, the hospital and unit components were not separable. For record count, the hospital component fell from 0.441 unrestricted to 0.321 at an hourly floor. A negative-binomial mixed model gave latent-scale hospital and unit variance-partition coefficients of 0.348 (95% CI 0.252-0.457) and 0.112 (0.078-0.163), equivalent to 0.318 and 0.110 observed. A pooled bottom-decile rule agreed with hospital-specific rules on 52.5% (4726/9006) of flagged stays, with 6 hospitals supplying half the pooled decile. The hospital component for record count replicated at 0.322 for oxygen saturation and 0.333 for respiratory rate.

**Conclusions:**

Within a multisite nurse-charted stream, vital-sign record volume varied more across contributing sites than across care units within hospitals; for the interval-structure metrics, the site and unit components were not separable at this cohort size. Apparent cross-database differences were reproduced by source-table choice, and site-level variation remained after excluding the most evident contribution-completeness outliers. Comparative use therefore requires specifying the generating stream, enforcing the analytic window, and explicitly modeling site-level variation; the residual site component should not be interpreted as documentation practice alone.

## Introduction

Electronic health record (EHR) data are widely reused for quality measurement, risk prediction, phenotyping, and comparative analysis [1-3]. Counts and timing of recorded observations are attractive process measures because they can be derived directly from routine care data. Vital-sign record density—the number of charted observations per unit time—is one such measure: it may reflect monitoring continuity or care intensity, but its interpretation depends on how the records were generated.

That property is also its principal hazard. What is recorded in an EHR reflects patient physiology, the clinical response to it, and the documentation process that captures that response. These layers can vary across units, sites, and health systems, and any count of records may index all three. Informative presence and informative observation describe how the timing of encounters and decisions to measure can depend on the latent health state under study [4-6]. Documentation habits can materially affect EHR-derived quality measures [3]; completeness requires an explicit, task-dependent definition for secondary use [7,8]; sampling on data sufficiency can select sicker patients [9]; and health care processes rather than physiology can drive apparent signal in EHR data [10]. Data-quality frameworks for secondary use are well established [7,11], and the risk that a measure behaves differently across deployment environments is recognized as dataset shift [12]. Recent work has assessed completeness within a single institution [13], quantified multisource coherence across 4 countries in a 652-participant cohort [14], and characterized the effect of data quality on integration pipelines by injecting structured noise into Medical Information Mart for Intensive Care III (MIMIC-III) [15]. What remains unclear for a routinely computed intensive care unit (ICU) process measure is the level at which observed variation resides across many contributing sites.

If record density varies primarily across generating streams—nurse-charted vs monitor-derived capture—then stream characterization may be sufficient before comparative use. If it varies primarily across contributing sites, cross-site comparison requires explicit modeling of site. If it varies primarily across care units, the measure is primarily a unit-level construct. These possibilities are empirically distinguishable and imply different remedies.

Two adult ICU databases permit this distinction. Medical Information Mart for Intensive Care IV (MIMIC-IV) provides data from 1 academic center with 9 named ICU types, holding the institution and EHR approximately fixed while the care unit varies [16]. The eICU Collaborative Research Database (eICU-CRD) provides data from 208 hospitals on a common telehealth platform, holding the delivered research-data structure approximately fixed while the contributing site varies [17]. Together, they support a decomposition that neither database supports alone.

The analysis uses heart rate as the primary variable and repeats its central sections for oxygen saturation and respiratory rate, so that findings specific to one variable can be distinguished from properties of routinely charted vital signs.

We asked 3 questions. First, what does a pooled-item exposure actually count, and does the answer depend on the source table? Second, at which level—database, contributing site, or care unit—does record density vary? Third, how sensitive are temporal and prognostic patterns to the precise specification of the exposure and the analytic window? We do not interpret any patient-outcome association as a study finding, and the Discussion explains why these data do not support one.

## Methods

### Data Sources

MIMIC-IV version 3.1 provides EHR data for adult ICU admissions at Beth Israel Deaconess Medical Center from 2008 through 2022 [16,18]. Vital signs are stored in the chartevents table. eICU-CRD version 2.0 provides data for adult ICU stays at 208 US hospitals during 2014-2015 [17,19]. eICU-CRD stores vital signs in 2 tables: vitalPeriodic, which contains 5-minute medians of continuously monitored signals, and nurseCharting, which contains nursing-charting observations, entered directly or interfaced from the bedside electronic medical record. Both datasets were extracted. Access was obtained through PhysioNet with credentialed access under the applicable data use agreements. Reporting follows STROBE (Strengthening the Reporting of Observational Studies in Epidemiology) [20].

### Cohort

The MIMIC-IV frame is the version 3.1 icustays table of 94,458 ICU stays, from which 9216 stays that were not the first ICU stay of their hospital admission were removed, together with 14 rows lacking valid admission or discharge time stamps, leaving the 85,228 stays at the head of the participant flow. The analytic unit is therefore the first ICU stay of a hospital admission, and ICU readmissions within the same hospitalization are not represented. No equivalent restriction is applied in eICU-CRD; its frame of 200,859 unit stays includes repeat unit admissions within a hospitalization. eICU-CRD does provide patientHealthSystemStayId, which links unit stays within a hospital admission, but the archived analysis was conducted at the unit-stay level throughout. The analytic unit therefore differs between the 2 cohorts. No age restriction was applied; MIMIC-IV comprises adult admissions only, and no length-of-stay restriction was applied at this stage. Removed and retained stays were similar in age (median 67, IQR 56-77 vs median 66, IQR 55-77 years), while removed stays had a longer ICU length of stay (median 2.56, IQR 1.28-5.26 vs median 1.92, IQR 1.08-3.73 days) and were modestly overrepresented in surgical and trauma units and in urgent admissions, as expected of readmissions within an admission; the exclusion rate ranged from 8.8% to 11.2% across the large care units and is therefore not strongly differential by unit. All adult ICU stays with at least 3 heart rate records in the first 24 hours were eligible. Timing metrics were computed for stays that completed the full 24-hour window (MIMIC-IV ICU length of stay ≥1 day; eICU unit discharge offset ≥1440 minutes), because stays ending earlier cannot generate a complete interval series and their gap statistics are truncation-biased. Intervals are the successive differences of the in-window record sequence, so a stay with n records yields n–1 intervals and the interval metrics, including the longest interval and the fraction of the window inside gaps, exclude both the time from ICU or unit admission to the first record and the time from the last record to the close of the window. Where the first record is delayed, this omission is material: in the cardiac vascular ICU the median time to first record is 2.57 hours, which the gap metrics do not count. Both the unrestricted and restricted analyses are reported where the distinction is material.

The 3-record minimum is necessary for interval statistics but conditions eligibility on the record-generating process under study. For the count end point, the analysis was therefore repeated for every stay completing the window, assigning a count of 0 where no heart rate record was present, and the proportion excluded by the criterion was tabulated by cohort and by hospital (see “Sensitivity Analyses” section). For stream-specific summaries, the 3-record criterion was applied within each source stream separately, yielding 119,317 eligible nurse-stream and 130,294 eligible monitor-stream eICU-CRD stays; paired nurse-vs-monitor analyses were restricted to the 117,572 stays meeting eligibility in both streams.

Analysis sets are distinguished throughout. The variance decomposition is reported in full on the restricted primary cohort: the pooled decomposition uses 155,700 stays across 61 hospitals (MIMIC-IV plus the 60 retained eICU-CRD hospitals), and the eICU-CRD η^2^ and variance-partition columns use the 88,560 eICU-CRD stays across those 60 hospitals, with 500 hospital-clustered bootstrap replicates for the η^2^ column. The corresponding quantities without the plausibility floor, reported as sensitivity analyses, are 186,457 pooled stays across 194 hospitals with 282 hospital-by-unit-type cells, 119,317 eICU-CRD stays across 193 hospitals for clustered inference across every hospital contributing eligible stays, and 96,518 stays across the 68 hospitals contributing at least 500 eligible stays for the mixed model and per-hospital summaries. Unit-level profile comparisons use hospital-by-unit-type cells with at least 50 stays: 238 eICU-CRD cells and 10 MIMIC-IV cells, the latter comprising the 9 named ICU types and a residual Other category (229 stays). The per-unit temporal models use the 9 named units with at least 800 stays. A minimum of 50 stays was selected for unit-level profile summaries; thresholds were fixed before the comparisons were run. The term unrestricted cohort is used throughout for the 96,518 eICU-CRD stays across the 68 hospitals contributing at least 500 eligible stays, the set that differs from the restricted primary cohort only by the plausibility floor; analyses requiring complete hospital attributes use the 86,395 stays across the 59 of those hospitals for which all 3 attributes are populated, against 78,437 stays across 51 hospitals in the restricted primary cohort.

### Exposure Definition and Composition

The exposure is the number of heart rate observations during the first 24 ICU hours. The comparator examined here, referred to throughout as the pooled-item count, was not drawn from a published study: it was defined by counting all heart rate–labeled items returned by a dictionary search of d_items. We report its composition because that pooling is the definition a researcher would reach without inspecting the charting profile of each item, and because it reconstructs exactly, so the consequences of the composition error can be quantified rather than asserted. Because the pooled-item count could not be reproduced from a single item identifier, we searched d_items for all heart rate–related items, counted each per stay within the window, and tested which combination reproduced the pooled-item count. Records were retained if the numeric value was between 20 and 250 beats per minute.

For eICU, the exposure was computed separately from vitalPeriodic (heartrate) and from nurseCharting (heart rate value label), enabling direct comparison of the 2 source tables across the same admissions.

### Replication in 2 Additional Vital Signs

To determine whether the findings are specific to heart rate documentation or to routinely charted vital signs, the central analyses were repeated for peripheral oxygen saturation and respiratory rate. MIMIC-IV used item IDs 220277 and 220210, respectively; eICU used the nurseCharting value names O_2_ Saturation and Respiratory Rate and the vitalPeriodic columns sao2 and respiration, all verified against the source dictionaries before extraction.

Alarm-limit and desaturation-limit items were excluded throughout (223769, 223770, and 226253 for oxygen saturation; 224161 and 224162 for respiratory rate), on the same grounds as for heart rate: each appears at approximately 2.8 records per stay, with 53% (274,880/518,527) of records falling within 3 clock hours, vs 13% (311,822/2,388,252) for the corresponding observation items. Ventilator-derived respiratory rate (224688, 224689, and 224690) was also excluded, appearing at 4.5 records per stay across approximately 35,000 stays on a distinct charting profile. It is a device stream, and combining it with a nurse stream would reproduce the source-stream mismatch examined in this study. That exclusion removes the principal charting mechanism for mechanically ventilated patients, so the respiratory-rate replication is weighted toward patients who are not mechanically ventilated, and its cohort is not equivalent in acuity to the heart rate cohort. Because the replication tests whether the variance structure is specific to one variable rather than estimating a population quantity, this shifts the population described rather than invalidating the comparison, but the replication should be read as covering nurse-charted respiratory rate in predominantly nonventilated patients. Four analyses were replicated: stream concordance within eICU-CRD, the between-database η^2^ under each pairing, hospital vs unit-within-hospital variance in the nurse streams, and MIMIC-IV position in the eICU-CRD unit distribution. Outcome, admission-hour, and threshold analyses were not repeated.

The 3-record minimum and the completed 24-hour window were applied separately to each variable, so the replication cohorts are variable-specific: 106,028 eICU-CRD nurse-stream stays for oxygen saturation and 114,258 for respiratory rate, compared with 119,317 for heart rate, contributed by 181, 190, and 193 hospitals, respectively.

### Plausibility Restriction

Hospital-level variation in a nurse-charted measure may reflect documentation practice or incomplete contribution of the stream to the research database, and the two support different conclusions. The observed cadence in both cohorts supplies an empirical reference: the median charting interval is 60 minutes in MIMIC-IV, and in the eICU-CRD nurse stream alike, so approximately hourly observation characterizes the pooled nurse-charted data in both cohorts. A hospital whose median stay records only a handful of heart rate values across 24 hours is therefore an order of magnitude below the observed norm, which is more consistent with partial contribution of the stream than with charting at that density.

The primary analysis excludes hospitals whose median stay records fewer than 12 heart rate observations in the first 24 hours, corresponding to 2-hourly observation. The floor is set below rather than at the approximately hourly cadence observed in both cohorts, and is therefore a conservative plausibility floor rather than a clinical standard in its own right; it was chosen for that reason rather than to maximize or minimize any estimate. Floors of 6 and 24 observations, corresponding to 4-hourly and hourly observation, are reported alongside. The unrestricted analysis is retained as a sensitivity analysis. For the replication variables, the floor is applied using each variable’s own median count, because a hospital may contribute one nurse-charted variable and not another. MIMIC-IV data come from a single institution, and no care unit falls below the floor, so no MIMIC-IV stay is excluded, and its cohort is identical under both analyses.

The provenance of this restriction is stated openly. It was not prespecified. It was introduced during peer review, in response to the observation that the sparsest contributing hospitals might not be contributing the nurse-charted stream in full, and the requested analysis showed that this concern was consequential: removing those hospitals reduced the hospital component for the median charting interval from 0.763 to 0.251 and for the fraction of the window inside gaps from 0.621 to 0.208. Because the requested analysis materially altered the estimates, the plausibility-restricted cohort is used for the principal estimates and the unrestricted cohort is retained, with the alternative floors, as sensitivity analyses. Primary here denotes the estimate less influenced by the most evident contribution-completeness outliers.

### Documentation Metrics

For each stay, we computed the record count; the hours from ICU admission to the first record; the median and IQR of intervals between consecutive records; the longest interval; counts of intervals exceeding 30 minutes and 2 hours; and the fraction of the 24-hour window that fell within intervals longer than 2 hours. For MIMIC-IV, we additionally computed the share of records charted within the first 5 minutes of a clock hour, as a marker of protocol-anchored charting; the eICU offsets are recorded relative to unit admission, so this metric was computed for MIMIC-IV only. An approximate clock time for each eICU record could be reconstructed by adding the record offset to the unit admission clock time in patient.unitadmittime24, which the admission-hour analysis already uses. The derivation was not part of the analysis plan; a site-level on-the-hour fraction obtained this way would bear on the default flowsheet cadence discussed below.

A record is a single time-stamped row in the source table carrying a value inside the plausible physiological range: 20 to 250 beats per minute for heart rate, 50% to 100% for oxygen saturation, and 4 to 60 breaths per minute for respiratory rate. Operational stream provenance, source-field selectors, record definitions, duplicate handling, and derived-metric definitions are provided in Multimedia Appendix 1. Rows sharing an identical time stamp for the same variable and stay were not collapsed, so simultaneous entries are counted separately and contribute zero-length intervals to the interval statistics. We verified that this is equivalent to counting distinct time stamps in these data. Among 2,444,918 MIMIC-IV heart rate records, no time stamp was duplicated within a stay; in eICU-CRD, 2 of 5,223,657 nurse-charted records and 17 of 45,164,349 monitor records were duplicated. Zero-length intervals accounted for under 0.001% of intervals in every stream, and no reported metric changed when identical time stamps were collapsed.

### Variance Decomposition

#### Estimators and Inference

Variance in each metric was summarized descriptively by database, hospital, and care unit using η^2^ computed from group sums of squares, reported as nested increments under the database-first ordering: the database-first increment, the subsequent hospital increment, and the unit-within-hospital increment. A density-conditional version was obtained by residualizing each metric on the log record count using least squares before decomposition, isolating differences in the shape of documentation from differences in its volume.

Because η^2^ from nested sums of squares is sensitive to unequal group sizes, and eICU-CRD hospital sizes are highly unequal, the decomposition was repeated as a linear mixed model with random intercepts for hospital and for unit within hospital, with metrics standardized before fitting. Variance partition coefficients are reported alongside the η^2^ estimates (see “Sensitivity Analyses” section).

Uncertainty was estimated by resampling hospitals with replacement, using 500 replicates, and recomputing the full decomposition within each replicate; hospitals drawn more than once were relabeled so they contributed as distinct clusters. η^2^ intervals are percentile intervals of that bootstrap distribution. Variance partition coefficients from the Gaussian models are reported with bias-corrected and accelerated intervals from the same hospital-clustered resampling, the acceleration estimated by a delete-one-hospital jackknife, because a percentile interval is miscentered for a ratio of variance components estimated from this many clusters: in simulation at the size and cluster structure of this cohort it covered the generating value 83% of the time against 93% for the bias-corrected interval, at equal width. The restricted maximum likelihood (REML) objective for this nested random-intercept model was evaluated in closed form from per-unit counts and sums rather than by general-purpose optimization, which is what makes 500 clustered replicates affordable for every metric; across the 16 point fits the 2 routes agreed to within 0.003, and the closed-form evaluation reached an REML objective no worse than the general-purpose optimizer’s in every case. The comparison is archived with the code. Coefficients from the negative binomial and zero-truncated negative binomial models are reported with profile-likelihood intervals, obtained by making the coefficient a free parameter and remaximizing the remaining parameters at each fixed value, because a clustered bootstrap of a generalized linear mixed model is not feasible at this scale. Because MIMIC-IV comprises a single institution, the database-first increment cannot be assigned a sampling distribution by this procedure and is reported as a point estimate; the hospital and unit components, which carry the substantive claim, were bootstrapped within eICU by resampling each hospital that contributed eligible stays. MIMIC-IV’s between-unit component was bootstrapped over stays.

#### Hospital Attributes

eICU-CRD records 3 hospital attributes in its hospital table, collected by self-reported survey: bed-capacity category (3 levels), teaching status (2 levels), and region (4 levels). We tested whether these account for the estimated hospital component in 5 principal documentation metrics: record count, median charting interval, longest interval, gaps exceeding 2 hours, and the fraction of the 24-hour window within gaps. Analyses use the complete-case hospital set, that is, hospitals for which all 3 attributes are populated, so that the conditional and unconditional estimates are computed on identical rows.

Each metric was regressed at stay level on indicator variables for the 3 attributes by least squares, giving 6 indicator coefficients after one level of each attribute was used as the reference category, and the hospital η^2^ was recomputed on the residuals. The reduction from the unconditional value is reported as the share of the hospital component accounted for by the recorded attributes. Because conditioning on 6 indicator coefficients removes part of a between-hospital structure by construction, each reduction was referred to an empirical null: the joint attribute vector was permuted across hospitals 10,000 times, holding each hospital’s stays together and the joint distribution of the 3 attributes fixed, and the reduction recomputed within each permutation. The empirical *P* value is 1 plus the number of permutations whose reduction equaled or exceeded the observed one, divided by 1 plus the number of permutations, so that the minimum attainable value is 1/10,001 at the 10,000 permutations used. The procedure was applied to the restricted primary cohort and repeated in the unrestricted cohort, giving 10 tests in total, to which a Bonferroni correction was applied at a threshold of .005.

### Distributional Assumptions and Entry Order

Because record count is a right-skewed nonnegative integer, a Gaussian model is not the appropriate distributional assumption for that outcome. The variance partition for record count was therefore also obtained from a negative binomial generalized linear mixed model with a log link, random intercepts for hospital and for unit within hospital, and a dispersion parameter estimated jointly with the variance components. The model was fitted by maximum likelihood using a Laplace approximation to the integral over the random effects, with the random effects profiled out by Newton iteration and the log-determinant of the penalized Hessian obtained from a sparse factorization. Variance partition coefficients are reported on the latent scale, in which the level-1 variance on the log link is ln(1 + 1/μ + α) for a negative binomial model with mean μ and dispersion α, and ln(1 + 1/μ) for a Poisson model, with μ being the cohort mean of the metric. The same specification was applied to the count of intervals exceeding 30 minutes, which is also a nonnegative integer. The count of gaps exceeding 2 hours is predominantly 0 and was fitted instead as a hurdle model in 2 parts: a Bernoulli generalized linear mixed model with a logit link for whether any such gap occurs, whose level-1 variance is π^2^ over 3, and a zero-truncated negative binomial generalized linear mixed model for how many occur given at least one, with the same random-effect structure; a hurdle model has one variance partition per part and no pooled coefficient, and both parts are reported. Where the dispersion of a zero-truncated fit fell to the boundary, it was held at 0.001 and the fit reported as indistinguishable from the equidispersed case. Because latent-scale coefficients are not comparable with the standardized observed-scale coefficients of the Gaussian models, an observed-scale equivalent is reported alongside every negative binomial fit, obtained in closed form from the lognormal moments of the random effects and verified by simulation from the fitted model. Before use, the estimator was verified on simulated overdispersed counts matched to the cohort in size, cluster structure, and variance-to-mean ratio, recovering the generating hospital variance to within 0.006, the unit variance to within 0.001, and the dispersion to within 0.0004.

The pooled decomposition uses sequential sums of squares and therefore depends on the order in which factors are entered. Because the 3 factors are perfectly nested, the contribution of each factor entered last is 0 by construction, and the sequential values span the full range across orderings. This was verified rather than assumed: entered last, both database and hospital contribute exactly 0.000 for every metric, and across orderings the hospital component for median charting interval ranges from 0.000 to 0.759. The database range of 1.6% to 7.1% reported below is therefore a property of entering the database first and is not an attribution of variance to the database level. The pooled columns are reported as a descriptive summary under a stated ordering rather than as an attribution of variance; the variance partition coefficients, which do not depend on entry order, carry the inferential statement.

### Stream Comparison

For eICU-CRD stays meeting eligibility in both source tables, we compared bottom-decile membership between the monitor-derived and nurse-derived counts. As in the “Consequence of a Global Threshold” section, record counts are integers, and a percentile rule includes all stays tied at the cutoff, so neither rule flags exactly the nominal percentile. The nurse stream, with counts near 28, has more ties than the monitor stream at near 280. The comparator for chance agreement is therefore the observed nurse-stream marginal rather than the nominal percentile, and agreement is reported as the ratio of observed to chance. The Jaccard index is also reported. We further computed the between-database η^2^ under each pairing of MIMIC-IV against an eICU-CRD stream and MIMIC-IV’s percentile position within the distribution of eICU-CRD unit cells under each pairing.

### Consequence of a Global Threshold

To express the variance decomposition as a consequence of the proposed use of record density, low density was designated at the 5th, 10th, and 20th percentiles in 2 ways: on the pooled distribution across all hospitals and within each hospital separately. Because record counts are integers and the rule includes all stays at or below the cutoff, the 2 rules do not flag identical numbers of stays; the within-site rule flags more because narrower within-site distributions place more stays at the cutoff value. All quantities are derived from a single contingency table: positive agreement is the share of pooled-flagged stays also flagged within site; the Jaccard index is the intersection over the union of the 2 flagged sets; and the reclassified share is the proportion of all stays flagged by one rule and not the other. Disagreement quantifies the extent to which designation depends on site-level differences rather than on within-site relative record density. The identical procedure was applied across the 9 named MIMIC-IV care units as an internal contrast.

### Admission-Hour Analysis

Bottom-decile membership was fitted as a logistic regression on 2-hour admission bins (reference 18:00-20:00), with adjustments for case mix, era, and care unit in MIMIC-IV and for age, sex, and unit type with hospital-clustered SEs in eICU-CRD. In MIMIC-IV, 4 specifications crossed exposure definition (pooled-item count vs observation-only count) with analytic window (all stays vs completed 24 hours). In eICU-CRD, 4 specifications crossed source stream (vitalPeriodic vs nurseCharting) with analytic window.

MIMIC-IV models adjusted for age, sex, comorbidity count, *International Classification of Diseases* chapter count, admission era group, and care unit; eICU-CRD models adjusted for age, sex, and unit type with SEs clustered by hospital. The per-unit MIMIC-IV models adjusted for age, sex, and comorbidity count, care unit being the stratifying variable.

All admission-hour models were fitted on complete cases; no covariate was imputed, and none was warranted at the observed rates. In MIMIC-IV, the admission-hour frame is the 85,228-stay cohort less the 95 stays carrying no heart rate record in chartevents, giving 85,133 stays, the same denominator on which the exposure reconstruction was verified; across those stays, all 6 covariates were complete. In eICU-CRD, the admission-hour models are fitted on the 177,198 unit stays present in both source streams rather than in either alone, so that the monitor-stream and nurse-stream specifications are estimated on identical rows and stream availability cannot confound the comparison between them. Within that frame, age was missing for 10 stays and sex for 18, 6 of them missing both, so listwise deletion removed 22 (0.012%) stays, leaving 177,176; unit type and unit admission clock time were complete, and 121,891 of the retained stays completed the 24-hour window. The nurse-stream frame alone comprises 181,731 stays and is the denominator for the stream-specific eligibility figures reported in Sensitivity Analyses, not for these models. In MIMIC-IV, the covariates were drawn from the stay-level cohort table, and stays lacking an admission-hour bin, or either exposure count, were dropped before fitting. In eICU-CRD, stays lacking a recorded unit admission clock time, an age value, or a sex value were dropped, age being read from the patient table with the deidentified ceiling category recoded to 90 years. Because listwise deletion on these covariates removes stays for reasons plausibly related to a site’s data contribution, the admission-hour estimates carry the same site-selection caveat as the primary analyses.

### Severity Harmonization

To assess whether the density-acuity relationship could be compared across cohorts, we attempted to construct a harmonized severity instrument. We measured coverage of candidate components in both cohorts before fitting any model. Analyses were conducted in Python 3 using pandas, NumPy, SciPy, and statsmodels.

### Ethical Considerations

This study used previously collected, deidentified, credentialed-access data from MIMIC-IV [16,18] and eICU-CRD [17,19] in accordance with the applicable PhysioNet data use agreements. The MIMIC-IV research resource was reviewed by the Institutional Review Board of Beth Israel Deaconess Medical Center, which granted a waiver of informed consent and approved the data-sharing initiative (IRB #2001P001699). eICU-CRD was deidentified in accordance with the Health Insurance Portability and Accountability Act (HIPAA) Safe Harbor provisions; its deidentification schema was independently certified by a privacy expert (Privacert; HIPAA Certification number 1031219-2), and the public database contains no retained key permitting the investigator to link its identifiers to identifiable individuals. No new patient data were collected, no participants were contacted, and the investigator had no means of reidentifying individuals.

Under University of Oxford Central University Research Ethics Committee guidance, secondary analysis of previously collected anonymized, nontraceable data does not require ethics committee scrutiny where the research team holds no reidentification code or other information permitting identification [21]. Accordingly, no separate ethics application was submitted for the present secondary analysis, and no additional informed consent was required.

## Results

### Exposure Composition

A dictionary search of d_items for heart rate–related items returned 24 candidates, of which 3 carry a heart rate label and appear at material volume: the observation item (220045) and the high and low alarm-limit items (220046 and 220047). The alarm items are documentation events rather than observations and are charted on a separate schedule (Table 1; Figure 1), so any exposure built by summing label-matched items mixes the two. The sum of the 3 reproduced the pooled-item count with 99.3% exact agreement across the 85,133 stays carrying both definitions, with a mean residual of +0.01 records, identifying that pooling as the definition is what such a search yields; no other combination of the 24 candidates came closer.

Including the alarm items inflates the mean count from 25.9 (SD 9.59) to 31.5 (SD 10.25) and shifts the bottom-decile threshold from 17 to 22 records. The 2 definitions agree on bottom-decile membership for 76% of the flagged stays (Jaccard 0.76). An independent reconstruction of the observation-only count matched a separately maintained extraction with 99.3% exact agreement (Pearson *r*=1.0000), confirming that the discrepancy lies in item composition rather than extraction error.

**Table 1:** Charting profile of the Medical Information Mart for Intensive Care IV (MIMIC-IV) heart rate items returned by a dictionary search of d_items: retrospective data quality study, adult intensive care unit stays, first 24 hours, 1 US academic medical center, 2008-2022 (MIMIC-IV version 3.1)^a^.

Item	Item ID	Records/stay	Share in 3 busiest hours	Busiest hours
Heart rate	220045	25.92	0.131	12, 14, 16
Heart rate alarm—high	220046	2.82	0.528	00, 08, 20
Heart rate alarm—low	220047	2.82	0.528	00, 08, 20
Reconstructed pooled-item count (reconstruction frame, 85,133 stays)	N/A^b^	31.49	N/A	N/A

^a^The 2 alarm items are documentation events rather than observations and are charted on a separate schedule. Heart rate observations were nearly uniformly distributed throughout the day, with 13.1% (320,908/2,446,114) of records falling in the 3 busiest clock hours; the alarm items concentrated 52.8% (277,016/524,618) of their records in 3 hours—00:00, 08:00, and 20:00—consistent with common shift-boundary times (Table 1; Figure 1). Observations averaged 25.9 (SD 9.59) records per stay, and each alarm item averaged 2.8 (SD 1.18). A uniform distribution across the day corresponds to a 3-hour share of 0.125. The first 3 rows characterize the items as the dictionary search returns them and are therefore computed on the source table rather than on the reconstruction frame: each is the mean over all MIMIC-IV intensive care unit stays carrying at least one record of the item concerned, 94,361 stays for item 220045, 93,018 for 220047, and 93,012 for 220046. Because the 3 denominators differ, the 3 means are not additive, and their sum of 31.56 is not a per-stay quantity; dividing all 2,970,732 records by the common denominator of 94,361 stays gives 31.48. The final row is on a different and narrower frame: it is the mean pooled-item count over the 85,133 stays of the reconstruction frame, which carry both count definitions and on which the reconstruction was verified, the same denominator as the admission-hour models, and on that same frame the observation item averages 25.88 (SD 9.47) rather than the 25.92 shown above. The 2 frames are reported separately because the item profile is a property of the source dictionary while the pooled-item count is the exposure actually analyzed; no analysis in this paper sums the first 3 rows. The summed 3-item definition reconstructed the pooled-item count with 99.3% exact agreement.

^b^N/A: not applicable.

**Figure 1:**
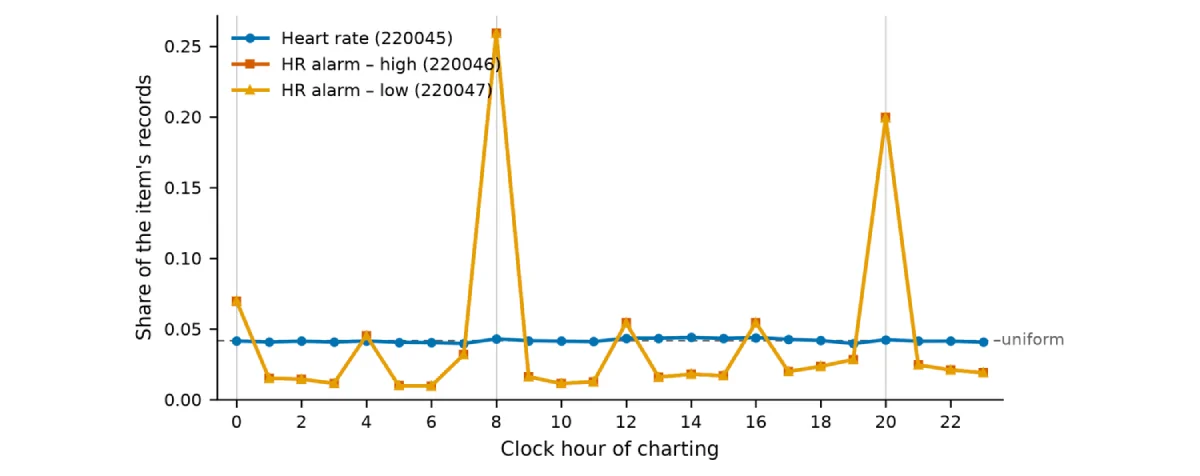
Hourly distribution of heart rate (HR) observations (item 220045) and heart rate alarm-limit records (items 220046 and 220047) charted during the first 24 hours of adult intensive care unit stays in Medical Information Mart for Intensive Care IV version 3.1, at 1 US academic medical center, 2008-2022. This retrospective data quality study found that observations were nearly uniform throughout the day. The 2 alarm-limit series are nearly superimposed and peak at 08:00 and 20:00, with a smaller peak at 00:00 and secondary peaks at 4-hourly intervals, consistent with common shift-boundary and shift-midpoint charting times.

### Source-Table Comparison

MIMIC-IV chartevents yielded a mean of 28.0 (SD 8.8) heart rate records per 24 hours (median 26, IQR 24-29; median interval 60, IQR 60-60 minutes). eICU nurseCharting yielded 34.9 (median 29, IQR 21-44; median interval 60, IQR 26.5-60 minutes). eICU vitalPeriodic yielded 273.5 (median 284, IQR 272-287; median interval 5, IQR 5-5 minutes). The nurse-charted streams share a cadence, whereas the monitor stream does not (Table 2).

Among 117,572 eICU-CRD stays present in both source tables—the same admissions, window, and physiological variable—the 2 counts were almost unrelated (Spearman ρ=0.024). The monitor rule flagged 10.2% (12,017/117,572) of stays, and the nurse rule 10.5% (12,367/117,572); of the monitor-flagged stays, 10.6% (1274/12,017) were also nurse-flagged, vs 10.5% (12,367/117,572) expected under independence, yielding an observed-to-chance ratio of 1.01 (Jaccard 0.055). At the 20th percentile, the ratio was 1.08, and at the 5th percentile, 0.86, the latter reflecting a nurse cutoff of 4 records, at which ties inflated that marginal to 0.060 vs 0.050 in the monitor stream. For heart rate, bottom-decile agreement was approximately what would be expected under independence.

The consequences for cross-database comparison are direct. Pairing MIMIC-IV with vitalPeriodic gave between-database η^2^ values of 0.960 for record count and 0.877 for median interval. Pairing the same MIMIC-IV data with nurseCharting reduced these to 0.026 and 0.016, respectively. Under the monitor pairing, every MIMIC-IV care unit fell at the 0th or 100th percentile of the eICU unit distribution across all metrics; under the nurse pairing, MIMIC-IV units fell between the 26th and 41st percentiles on record count and at the 37th on median interval (Table 2).

**Table 2:** Source stream determines the cross-database comparison of vital-sign record density: retrospective data quality study of adult intensive care unit stays in the first 24 hours, Medical Information Mart for Intensive Care IV (MIMIC-IV) version 3.1 (1 US academic medical center, 2008-2022) and eICU Collaborative Research Database (eICU-CRD) version 2.0 (208 US hospitals, 2014-2015).

Quantity	MIMIC-IV chartevents	eICU-CRD nurseCharting	eICU-CRD vitalPeriodic	Note
Stays analyzed	67,140	119,317	130,294	—^a^
Records/24 hours, mean (SD)	28.0 (8.8)	34.9 (24.3)	273.5 (28.5)	—
Records, median (IQR)	26 (24-29)	29 (21-44)	284 (272-287)	—
Interval (minutes), median (IQR)	60 (60-60)	60 (26.5-60)	5 (5-5)	—
Between-database η^2^ vs MIMIC-IV (count)	—	0.026	0.960	Same MIMIC-IV data
Between-database η^2^ vs MIMIC-IV (interval)	—	0.016	0.877	Same MIMIC-IV data
MIMIC-IV percentile (count)	—	26th-41st	0th	Across MIMIC-IV units
Bottom-decile concordance	—	0.106 (chance 0.105; ratio 1.01)	—	117,572 shared stays

^a^Not available.

### Site-Level Variation

A total of 8 of the 68 eICU-CRD hospitals contributing at least 500 eligible stays recorded a median of fewer than 12 nurse-charted heart rate observations per 24-hour stay. Their median counts were 3 (IQR 3-4), 3 (IQR 3-4), 3 (IQR 3-4), 4 (IQR 3-4), 4 (IQR 3-4), 6 (IQR 5-7), 7 (IQR 6-12), and 9 (IQR 7-26), against 32 for the cohort as a whole, and between 1.4% (20/1384) and 33.8% (663/1959) of their stays fell below the 3-record minimum, against a cohort median of 0.5% (IQR 0.1%-7.3%). Separately, among all hospitals contributing at least 100 stays, 6 excluded every stay under the 3-record minimum and 4 more excluded between 92% and 98%; those 10 supplied 26% of all excluded stays. Those sites are distinct from the 8 below the plausibility floor, which retain enough eligible stays to be estimated. Per-hospital median counts for heart rate and respiratory rate correlated at 0.973, with oxygen saturation at 0.610. This cross-variable concordance is consistent with a site-level contribution or completeness mechanism, while the weaker oxygen-saturation correlation indicates that the pattern is not uniform and does not exclude variable-specific charting differences. These hospitals were excluded from the primary analysis, leaving 60 hospitals and 88,560 stays (Figure 2).

In the pooled eligible-stay sensitivity set (186,457 stays; 193 eICU-CRD hospitals plus MIMIC-IV; 282 unit cells), for record count and interval-structure metrics other than intervals exceeding 30 minutes, the database-first increment was 1.6%-7.1% of variance, the subsequent hospital increment was 42.6%-74.3%, and the unit-within-hospital increment was 0.4%-2.8%. In the pooled restricted primary set, the corresponding increments were 1.7%-8.1%, 10.0%-29.4%, and 3.1%-5.1% (Table 3; Figure 3). Hours to first record showed a different pattern, with the unit increment exceeding the hospital increment: in the pooled restricted primary set, the database-first increment was 3.6%, the subsequent hospital increment was 1.9%, and the unit increment was 16.5%, the unit effect being concentrated in a single care unit with a distinct admission pathway (see “Unit-Level Variation Is Concentrated in One Care Unit” section). Intervals exceeding 30 minutes retained a large database-first increment in both pooled sets (24.9% in the pooled eligible-stay sensitivity set, 23.1% in the pooled restricted primary set) for the reason given in the “Site Distribution and Charting Regularity” section.

**Figure 2:**
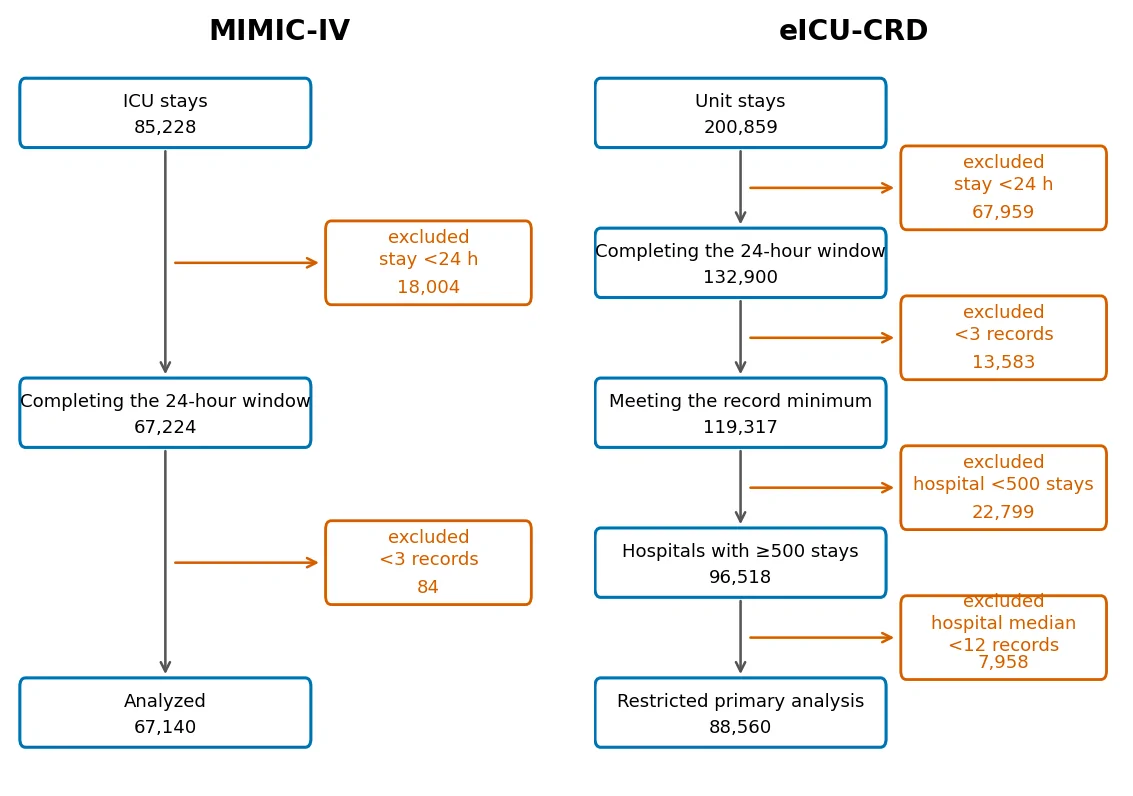
Participant flow for both cohorts of the retrospective data quality study of adult intensive care unit (ICU) vital-sign record density (Medical Information Mart for Intensive Care IV [MIMIC-IV] version 3.1, 1 US academic medical center, 2008-2022; eICU Collaborative Research Database [eICU-CRD] version 2.0, US hospitals, 2014-2015). The MIMIC-IV pathway begins with the 94,458 rows of the version 3.1 icustays table, from which 9216 ICU stays that were not the first of their hospital admission and 14 rows lacking valid admission or discharge time stamps were removed, leaving 85,228 stays at the head of the diagram; the analytic unit is therefore the first ICU stay of a hospital admission, and ICU readmissions within the same hospitalization are not represented. The admission-hour models do not apply the 24-hour window and form a parallel branch: 85,133 MIMIC-IV stays, being the 85,228 less 95 with no heart-rate record, and 177,198 eICU-CRD unit stays, being the 200,859 of the patient table less 23,661 not present in both source streams, of which 177,176 remain after listwise deletion for age and sex. The stream-specific sets are completed-window sets under a different stream eligibility rule, and branch from the 132,900 eICU-CRD stays completing the window: 130,294 have at least 3 in-window vitalPeriodic records, 2606 do not, and 117,572 are eligible in both source streams; 15,328 are not. Both cohorts otherwise exclude stays not completing the 24-hour window, then stays with fewer than 3 heart rate records. eICU-CRD then restricts to hospitals contributing at least 500 eligible stays and, for the primary analysis, to hospitals whose median eligible stay records at least 12 heart rate observations in the first 24 hours. MIMIC-IV: 67,224 stays completing the window, 84 excluded by the 3-record minimum (72 with no record, 12 with one or two), leaving 67,140. eICU-CRD: 132,900 stays completing the window, 13,583 excluded by the 3-record minimum (8934 with no record, 4649 with one or two), leaving 119,317 across 193 hospitals; 96,518 stays across 68 hospitals after the 500-stay threshold; and 88,560 stays across 60 hospitals after the plausibility floor.

**Table 3:** Heart rate documentation variance decomposition: retrospective data quality study, adult intensive care unit stays, first 24 hours, Medical Information Mart for Intensive Care IV version 3.1 (1 US academic medical center, 2008-2022) and eICU Collaborative Research Database (eICU-CRD) version 2.0 (US hospitals, 2014-2015), nurse-charted streams^a^.

Metric	Database-first	+ Hospital	+ Unit	eICU-CRD hospital, η^2^ (95% CI)	Hospital VPC^b^ (95% CI)
Record count	0.081	0.294	0.038	0.354 (0.259-0.450)	0.348 (0.252-0.457)
Hours to first record	0.036	0.019	0.165	0.043 (0.026-0.056)	0.025 (0.007-0.050)
Median interval	0.017	0.225	0.031	0.251 (0.149-0.362)	0.190 (0.068-0.359)
Interval IQR	0.028	0.102	0.051	0.140 (0.085-0.198)	0.077 (0.012-0.198)
Longest interval	0.036	0.100	0.042	0.134 (0.077-0.192)	0.083 (0.022-0.191)
Gaps >30 minutes	0.231	0.253	0.047	0.377 (0.268-0.481)	0.376 (0.264-0.494)
Gaps >2 hours	0.034	0.183	0.046	0.218 (0.127-0.305)	0.174 (0.044-0.335)
Fraction of window in gaps	0.035	0.179	0.043	0.208 (0.123-0.287)	0.157 (0.018-0.334)

^a^Pooled columns are sequential nested increments entered into the database first, hospital second, and unit within hospital third across the pooled restricted primary set of 155,700 stays and 61 sites (Medical Information Mart for Intensive Care IV [MIMIC-IV] plus the 60 retained eICU-CRD hospitals); the corresponding pooled eligible-stay sensitivity set is 186,457 stays across 194 sites. The eICU-CRD η^2^ and VPC columns are estimated on the restricted primary cohort of 88,560 stays across 60 hospitals; η^2^ uses 500 hospital-clustered bootstrap replicates with percentile intervals, and corresponding unit-within-hospital estimates ranged from 0.031 to 0.044. Analysis sets are named consistently throughout: the restricted primary cohort is 88,560 eICU-CRD stays across 60 hospitals, the unrestricted cohort is 96,518 stays across 68 hospitals, the eligible-stay set is 119,317 stays across 193 hospitals, and the pooled equivalents add MIMIC-IV; Multimedia Appendix 1 tabulates every set with its definition. Hospital VPC is from a 3-component random-intercept model with hospital and unit within hospital on the same cohort. The column mixes scales by row: the record-count and intervals-exceeding-30-minutes rows are latent-scale negative binomial coefficients, with their observed-scale equivalents given below, and the remaining rows are standardized observed-scale Gaussian coefficients. For record count and for intervals exceeding 30 minutes the coefficients are from fitted negative binomial generalized linear mixed models, because a Gaussian model is not the appropriate distributional assumption for a count outcome, and their intervals are profile-likelihood intervals; the corresponding Gaussian values are 0.306 and 0.350, and the negative binomial unit-within-hospital coefficients are 0.112 (95% CI 0.078-0.163) and 0.135 (0.092-0.202). These are latent-scale coefficients, in which the level-1 variance is ln(1 + 1/μ + α), with μ the cohort mean of the metric, 39.01 and 14.34, respectively; on the observed scale, the hospital coefficients are 0.318 and 0.343, and the unit coefficients are 0.110 and 0.135. For the continuous and interval-structure metrics the coefficients are from Gaussian linear mixed models with bias-corrected and accelerated hospital-clustered bootstrap intervals, and their unit-within-hospital coefficients were 0.030 (0.009-0.069) for hours to first record, 0.102 (0.038-0.216) for the median interval, 0.109 (0.062-0.175) for the interval IQR, 0.097 (0.048-0.159) for the longest interval, 0.099 (0.044-0.176) for gaps beyond 2 hours and 0.108 (0.040-0.198) for the fraction of the window inside gaps. For record count and intervals exceeding 30 minutes, the Gaussian hospital coefficients and their intervals, reported for comparison with the count models, are 0.306 (95% CI 0.204-0.450) and 0.350 (0.266-0.498), against unit coefficients of 0.105 (0.057-0.198) and 0.093 (0.058-0.135). Gaps exceeding 2 hours are 73.7% (65,301/88,560) zeros, and the appropriate specification is the hurdle model reported in the text, which has no single pooled coefficient; the value shown in this column for that row is therefore a Gaussian sensitivity estimate and is not the inferential estimate for the metric. All cells in this table were computed in a single run over the 8 metrics so that each has one provenance. On the unrestricted cohort of 96,518 stays across 68 hospitals, the corresponding hospital η^2^ values were 0.441 for record count, 0.763 for median interval, 0.470 for interval IQR, 0.586 for the longest interval, 0.468 for gaps beyond 2 hours, and 0.621 for the fraction of the window inside gaps.

^b^VPC: variance partition coefficient.

**Figure 3:**
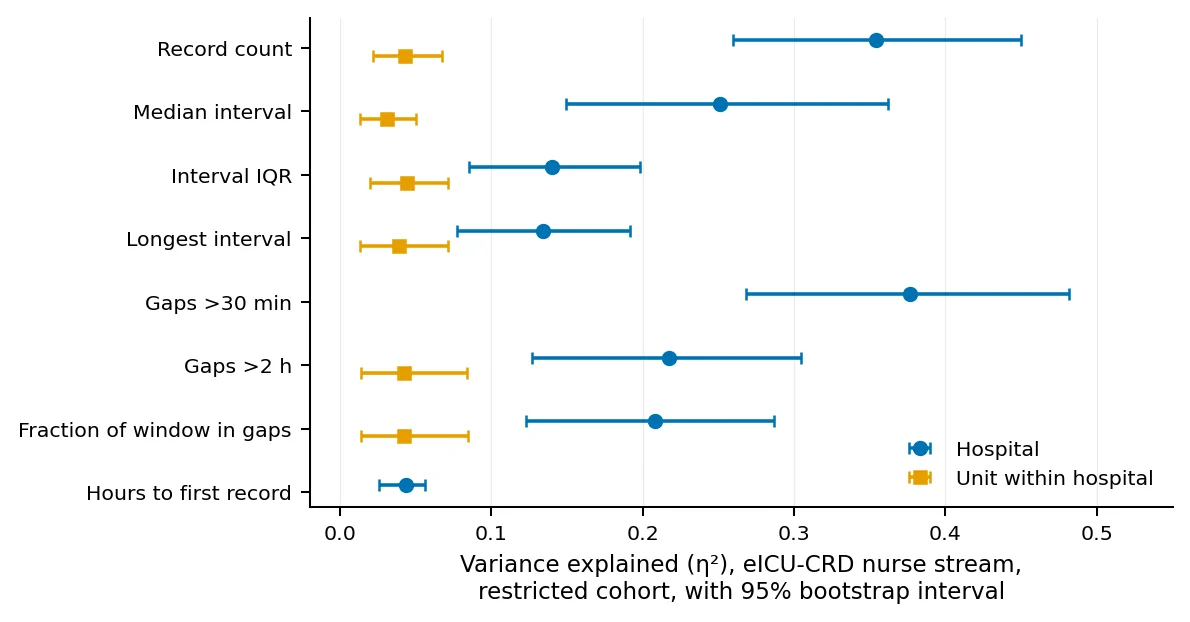
Hospital- and unit-within-hospital η2 increments for heart rate documentation metrics in the eICU Collaborative Research Database (eICU-CRD) version 2.0 nurse-charted stream, adult intensive care unit stays in the first 24 hours at US hospitals during 2014-2015; retrospective data quality study. The primary cohort was restricted to 60 hospitals and 88,560 stays, with 95% hospital-clustered bootstrap intervals for the hospital and unit η2 increments estimated within the eICU-CRD.

The database-first increment in Table 3 contrasts 1 academic medical center against a multisite collection. It therefore indexes the documentation policy of that center as much as any property of the database itself, and the distinction between data architecture and institutional policy cannot be drawn on the MIMIC-IV side of the comparison. The count of intervals exceeding 30 minutes deviated from the database-first pattern. In the pooled restricted primary set, the database-first increment was 23.1%, the subsequent hospital increment was 25.3%, and the unit increment was 4.7% (Table 3); in the pooled eligible-stay sensitivity set, the corresponding values were 24.9%, 33.2%, and 3.3%. The metric counts intervals in which an approximately hourly cadence was not met rather than interruptions of a denser one: 91.9% (1,268,105/1,379,521) of MIMIC-IV intervals exceed 30 minutes and 74.2% (1,168,014/1,574,690) of those in eICU-CRD fall between 55 and 65 minutes, so in both databases it largely reflects routine hourly charting. Within a fixed 24-hour window, it is therefore related to record count by construction, and the two move inversely: across the restricted primary cohort the Pearson correlation between them is –0.615 (*r*^2^=0.378) and the Spearman correlation –0.461, against –0.376 (0.141) and –0.225 in the unrestricted cohort. The count of intervals exceeding 30 minutes is accordingly a second view of charting density rather than an independent second outcome, and the 2 count results should be read together rather than as separate confirmations.

Within eICU, where clustered inference is available, hospital η^2^ in the restricted primary analysis was 0.251 (95% CI 0.149-0.362) for the median charting interval, 0.208 (0.123-0.287) for the fraction of the window inside gaps, 0.134 (0.077-0.192) for the longest interval, 0.218 (0.127-0.305) for gaps beyond 2 hours, and 0.354 (0.259-0.450) for record count. Within-hospital unit η^2^ ranged from 0.031 to 0.044. That ratio is not the ratio of variance components. Simulation from the fitted negative binomial model reproduces the observed η^2^ for record count (0.368, SD 0.043, against 0.354 observed) while the model’s own observed-scale hospital coefficient is 0.318 (95% CI 0.252-0.457), while for the unit component the simulated η^2^ of 0.059 (SD 0.015) lies below the model’s own coefficient of 0.110, and the observed value of 0.043 lies below the simulated one. η^2^ therefore overstates the hospital share and understates the unit share at 60 hospitals, inflating the ratio from both directions, so the order-free coefficients and their intervals carry the comparison. In the unrestricted sensitivity analysis, the corresponding hospital values were 0.763, 0.621, 0.586, 0.468, and 0.441, so approximately two-thirds of the apparent hospital component for the interval metrics was contributed by the 8 excluded hospitals. The plausibility floor was varied across 6, 12, and 24 observations per 24 hours, with the unrestricted analysis providing the no-floor comparison. With no floor, 68 hospitals contributed, and the hospital component was 0.441 for record count, 0.763 for median interval, and 0.621 for the fraction of the window inside gaps. At a floor of 6 observations, corresponding to 4-hourly observations, 63 hospitals remained, and the components were 0.386, 0.459, and 0.442. At 12 observations, or 2-hourly, 60 hospitals remained, and the components were 0.354, 0.251, and 0.208. At 24 observations, or hourly, 55 hospitals remained, and the components were 0.321, 0.188, and 0.149. The interval and gap components therefore decline steeply as probable incomplete contribution is removed, whereas record count declines by roughly a quarter across the full range; at every floor the hospital η^2^ increment remains the larger of the two, although η^2^ overstates that ordering for the reasons set out below.

A negative binomial generalized linear mixed model was fitted to the record count, appropriate because the counts were overdispersed, with a variance-to-mean ratio of 13.9 in the restricted cohort and 16.0 in the unrestricted cohort. In the restricted cohort, it converged with a hospital variance of 0.104, a unit-within-hospital variance of 0.034, and a dispersion of 0.151, giving latent-scale variance partition coefficients of 0.348 for hospital and 0.112 for unit within hospital, against 0.306 and 0.105 under the Gaussian model. In the unrestricted 68-hospital cohort, the corresponding hospital variance was 0.519 and the dispersion 0.150, giving variance partition coefficients of 0.728 for hospital and 0.043 for unit, against 0.434 and 0.083 in the Gaussian REML sensitivity analysis. Because the negative binomial coefficients are on the latent scale, where the level-1 variance is ln(1 + 1/μ + α), with μ the cohort mean record count of 39.01 (SD 23.3), and the Gaussian coefficients are on the standardized observed scale, the two are not directly comparable. Converted to the observed scale, which is exact for this model and confirmed by simulation, the negative binomial hospital coefficient is 0.318 in the restricted cohort against 0.306 under the Gaussian specification, and 0.668 against 0.434 in the unrestricted cohort. The site-dominant pattern is therefore not an artifact of the distributional assumption; in the primary cohort the count model leaves the site share essentially unchanged rather than increasing it. A Poisson specification, fitted by the same routine, placed the unrestricted hospital coefficient at 0.897 on the latent scale. The 2 families agree almost exactly on the hospital variance itself, 0.5174 against 0.5186, so the difference in the coefficient comes from the level-1 variance the Poisson assumes, 0.0271 against 0.1633; its log-likelihood is lower by 184,439, and the negative binomial is the appropriate family. Estimators of the unit component differed: sequential η^2^ placed it at 0.031-0.043, a within-hospital formulation at 0.041-0.066, the Gaussian variance partition coefficient at 0.103-0.107, and the fitted negative binomial model at 0.112. All 4 place it below 0.12, as do the hospital coefficients for hours to first record (0.025), the IQR (0.077) and the longest interval (0.083), so a unit coefficient below that level is not by itself evidence of a smaller unit component. Under the sequential η^2^ decomposition, the ratio of site to unit variation in the restricted cohort was approximately 3 to 8, rather than the order of magnitude seen before restriction; because η^2^ overstates the hospital share and understates the unit share at this number of clusters, that range is an upper bound on the ratio rather than an estimate of it. A total of 26 (43%) of the 60 hospitals in the restricted primary cohort contribute a single unit type, as do 30 (44%) of the 68 in the unrestricted cohort and 151 (78%) of the 193 hospitals contributing eligible stays. Where a hospital contributes one unit type, its unit variance cannot be separated from the hospital component, so the hospital coefficient is likely an upper bound and the unit coefficient likely a lower bound. Counts of intervals exceeding 30 minutes and of gaps exceeding 2 hours are also nonnegative integers and were refitted accordingly. For intervals exceeding 30 minutes, a negative binomial model gave hospital and unit coefficients of 0.376 (95% CI 0.264-0.494) and 0.135 (95% CI 0.092-0.202) on the latent scale, equivalent to 0.343 and 0.135 on the observed scale, reproducing the record-count pattern. Gaps exceeding 2 hours are 73.7% (65,301/88,560) zeros in the restricted cohort, so a hurdle specification was used: a Bernoulli model for whether any such gap occurs, giving coefficients of 0.136 and 0.089, and a zero-truncated negative binomial model for how many occur given at least one, giving 0.390 (95% CI 0.243-0.532) and 0.266 (95% CI 0.176-0.390) on the latent scale and 0.280 and 0.308 on the observed scale. A hurdle model has one variance partition per part and no pooled coefficient, so both are reported, and neither is presented as the coefficient for the metric. Profile-likelihood intervals are given for the zero-truncated part; the Bernoulli coefficients are reported as point estimates. On the observed scale, the unit coefficient of the count part is the larger point estimate, 0.308 against 0.280, and the latent-scale intervals overlap throughout, so site-level variation in gap structure is not distinguishable from unit-level variation in these data.

Conditioning on log record count left 0.323-0.458 of the hospital component intact, indicating that sites differ in the shape of documentation, not only in its volume. In the restricted cohort, per-hospital median charting intervals ranged from 15 minutes at the 10th percentile to 60 minutes at the 90th, a fourfold spread, against a twelvefold spread from 15 to 178 minutes before restriction.

### Within-Institution Variation

MIMIC-IV’s 9 named ICU types were closely similar: every unit had a median charting interval of 60 (IQR 60-60) minutes, a median longest interval of 60 (IQR 60-99) minutes, a median of zero gaps beyond 2 hours, and median record counts between 23 (IQR 19-25) and 27 (IQR 25-32). Between-unit η^2^ was 0.027 (95% CI 0.017-0.037) for record count and 0.039 (0.036-0.043) for median interval—the same order as the unit-within-hospital component estimated independently in eICU.

Two exceptions concerned timing rather than volume. Hours to first record varied from 0.22 to 2.57 across units (η^2^ 0.300, 95% CI 0.292-0.309), rising to 0.314 after conditioning on density; the cardiac vascular ICU accounted for the extreme, consistent with postoperative admission through the operating room. The share of records charted on the hour varied from 0.769 to 0.920 (η^2^ 0.071 raw, 0.174 conditional on density). For both metrics, the effect strengthened after adjustment for volume, indicating that units differ in when they chart rather than how much.

### Unit-Level Variation Is Concentrated in One Care Unit

The unit component within MIMIC-IV was not evenly distributed. Removing the cardiac vascular ICU alone eliminated 97.2% of the between-unit variance in hours to first record, 87.8% for the longest interval, 84.5% for the fraction of the window inside gaps, 80.0% for the interval IQR, and 62.1% for the on-the-hour charting fraction. That unit fell outside the range of all 8 others on 5 of 7 metrics, recording a median of 2.57 (IQR 1.08-4.09) hours to first observation against a maximum of 0.32 hours elsewhere.

This is consistent with its admission pathway: cardiac surgical patients arrive from theatre, where charting is maintained under a separate record until handover, so the first ICU observation is displaced rather than absent. Unit-level variation in these data is therefore not a general property of care unit but a property of one structurally distinct admission route, with the remaining 8 units closely similar.

### Site Distribution and Charting Regularity

Among the 238 eICU-CRD unit cells with at least 50 stays, MIMIC-IV units fell within the site distribution, at the 26th-41st percentile for record count, the 37th percentile for median interval, and the 23rd percentile for longest interval (Figure 4). They fell at the 0th percentile for interval dispersion and gap metrics: MIMIC-IV stays showed an interquartile interval range of zero and no gaps beyond 2 hours, whereas eICU-CRD unit medians ranged up to 61 minutes and 2 gaps, respectively. In matched unit-type comparisons, which reduce gross unit-type heterogeneity but do not establish acuity equivalence, the same pattern held for every mapped pair. Unit nomenclature mapped between 1 tertiary academic center and a diverse multihospital network does not guarantee comparable patient acuity, and neither database records nurse-to-patient ratio. Teaching status and bed-capacity category are recorded in eICU-CRD and are examined below; nurse-to-patient ratio is not recorded, and could drive documentation differences independently of the mechanisms discussed here from inside the hospital component.

The mechanism is evident in the interval distribution. Of MIMIC-IV intervals exceeding 30 minutes, 91.9% (1,268,105/1,379,521) fell between 55 and 65 minutes, and the 10th through 90th percentiles were all exactly 60 minutes; the corresponding eICU figures were 74.2% (1,168,014/1,574,690) and a 90th percentile of 120 minutes. The metric therefore counts routine hourly charting in MIMIC-IV and more irregular or prolonged intervals in eICU-CRD, which accounts for its anomalous database-first increment in Table 3. MIMIC-IV’s distinguishing feature is a highly regular approximately hourly charting cadence, not greater charting intensity.

**Figure 4:**
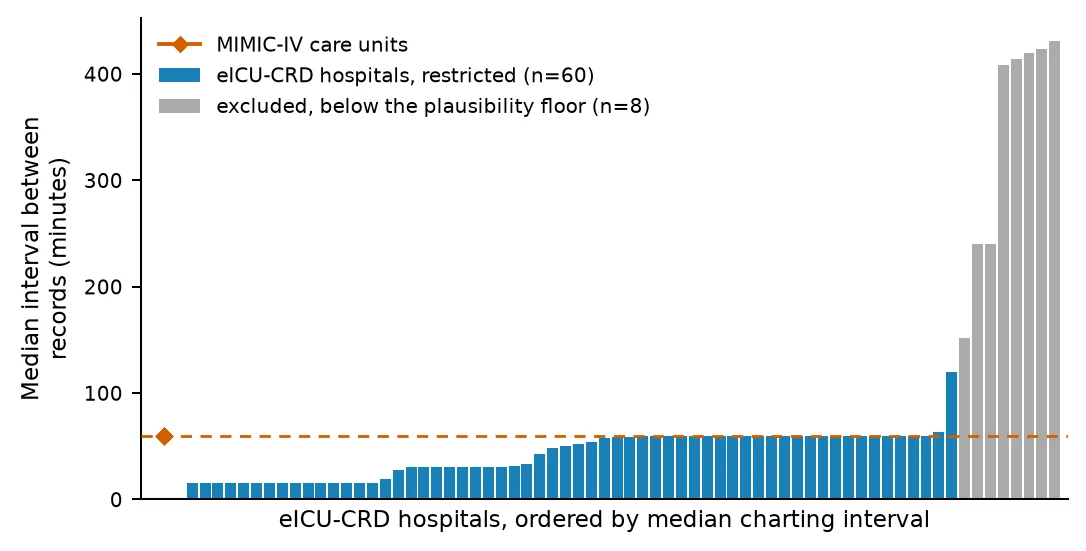
Distribution of the per-hospital median heart rate charting interval across 68 eICU Collaborative Research Database (eICU-CRD) version 2.0 hospitals contributing at least 500 eligible adult intensive care unit stays (nurse-charted stream, first 24 hours, US hospitals, 2014-2015); retrospective data quality study. The 60 hospitals retained in the primary analysis are shown in blue, and the 8 excluded as below the plausibility floor are shown in gray. The 9 Medical Information Mart for Intensive Care IV (MIMIC-IV) care units are overlaid; all 9 share a median interval of 60 minutes and therefore coincide as a single marker. MIMIC-IV sits within the site distribution on cadence while showing no within-stay interval dispersion.

### Admission-Hour Analysis

Fitted on all stays, low record density peaks in admissions occurred between 04:00 and 06:00, with an adjusted odds ratio (OR) of 3.34 (95% CI 3.00-3.72) on the pooled-item count and 3.67 (95% CI 3.29-4.09) on the observation-only count. Restricting to stays completing the 24-hour window reduced the corresponding estimates to 0.99 (95% CI 0.89-1.10) and 1.25 (95% CI 1.13-1.38), respectively (Table 4).

Exposure composition therefore had little influence on this result; the analytic window had a large influence. Admission hour predicts short stays, and a stay ending before 24 hours yields fewer records by definition. The association was not abolished by restriction: the observation-only estimate of 1.25 remained distinguishable from unity, and within the completed window the cardiac vascular ICU retained an association (OR 1.77, 95% CI 1.32-2.38), while the 8 other MIMIC-IV units were null.

In eICU, the association was present in the monitor stream (OR 1.61, 95% CI 1.39-1.86 across all stays; OR 1.41, 95% CI 1.18-1.69 restricted) and absent in the nurse stream (OR 1.08, 95% CI 0.98-1.20 across all stays; *P*=.11; OR 1.03, 95% CI 0.92-1.15 restricted; *P*=.66). In the admission-hour peak set, the 71 hospitals contributing at least 500 window-completing stays to the eICU-CRD admission-hour frame and 99,047 stays in all, 69% (49/71) showed peak low-density admission in the 00:00-14:00 window in the nurse stream.

**Table 4:** Association between intensive care unit (ICU) admission hour and bottom-decile record density under 4 specifications crossing exposure definition with analytic window: retrospective data quality study of adult ICU stays, Medical Information Mart for Intensive Care IV (MIMIC-IV) version 3.1 (1 US academic medical center, 2008-2022) and eICU Collaborative Research Database (eICU-CRD) version 2.0 (US hospitals, 2014-2015)^a^.

Cohort/stream	Exposure	Window	Peak bin	Adjusted OR^b^ (95% CI)
MIMIC-IV chartevents	Pooled-item count	All stays	04:00-06:00	3.34 (3.00-3.72)
MIMIC-IV chartevents	Pooled-item count	Completed 24 h	06:00-08:00	0.99 (0.89-1.10)
MIMIC-IV chartevents	Observations only	All stays	04:00-06:00	3.67 (3.29-4.09)
MIMIC-IV chartevents	Observations only	Completed 24 h	08:00-10:00	1.25 (1.13-1.38)
eICU-CRD vitalPeriodic	Monitor stream	All stays	14:00-16:00	1.61 (1.39-1.86)
eICU-CRD vitalPeriodic	Monitor stream	Completed 24 h	14:00-16:00	1.41 (1.18-1.69)
eICU-CRD nurseCharting	Nurse stream	All stays	16:00-18:00	1.08 (0.98-1.20)
eICU-CRD nurseCharting	Nurse stream	Completed 24 h	16:00-18:00	1.03 (0.92-1.15)

^a^Reference bin: 18:00-20:00. eICU-CRD models use hospital-clustered SEs. Models are complete-case with no imputation; covariate missingness removed 0 of 85,133 MIMIC-IV stays and 22 of 177,198 (0.012%) eICU-CRD stays. The eICU-CRD models are fitted on the stays present in both source streams, so the monitor-stream and nurse-stream rows are estimated on identical rows.

^a^OR: odds ratio.

### Severity Harmonization

Candidate components of a harmonized severity instrument were unevenly recorded. Serum bilirubin was present for 43.5% (41,126/94,458) of MIMIC-IV stays and 37.1% (74,584/200,859) of eICU-CRD stays in the first 24 hours; arterial oxygen tension for 54.0% (50,986/94,458) of MIMIC-IV stays; inspired oxygen fraction for 33.1% (66,532/200,859) of eICU-CRD stays; urine output for 49.1% (84,062/171,177) of eICU-CRD stays; and elective-surgery status for 19.4% (33,256/171,177). The Acute Physiology and Chronic Health Evaluation (APACHE) physiology table was unavailable for 9.7% of eICU-CRD stays in the restricted primary cohort; those missing APACHE stays were approximately fivefold enriched in the exposure bottom decile (38.3% vs 7.1%). Restricting analyses to stays with APACHE data would therefore induce exposure-related selection.

The components with adequate coverage—heart rate, mean arterial pressure, respiratory rate, temperature, and Glasgow Coma Scale—were derived from the same nurse-charting process that generates the exposure and therefore could not separate documentation intensity from patient acuity. Point estimates for the density-mortality association varied with the adjustment instrument: in eICU-CRD, the bottom-decile OR was 0.658 (95% CI 0.613-0.706) under minimal adjustment, 1.375 under APACHE-IV adjustment, and 1.111 (95% CI 0.995-1.241) under a harmonized vital-sign instrument. We therefore do not interpret any outcome association as a study finding.

### Sensitivity Analyses

#### Eligibility Criteria

Of stays completing the 24-hour window, 0.12% of MIMIC-IV stays were excluded by the 3-record minimum (72 with no records and 12 with one or two, of 67,224). In eICU, the corresponding figure was 10.22% (13,583/132,900) of 132,900 stays: 8934 (6.72%) had no nurse-charted heart rate record, and 4649 (3.50%) had one or two. The exclusion share varied markedly across hospitals (median 0.005, 10th percentile 0.000, 90th percentile 0.352, among the 80 hospitals contributing at least 500 window-completing stays, 108,305 stays in all, with stays carrying no nurse-charted heart rate record counted as zero) and correlated negatively with a hospital’s median count of eligible stays (Spearman ρ=–0.538), indicating that sparsely charting sites lose disproportionately many stays to the criterion.

The criterion therefore attenuated rather than produced the between-hospital component. Within those same 80 hospitals, that is, the hospitals contributing at least 500 window-completing stays on the 132,900-stay denominator, hospital η^2^ for record count was 0.510 when stays with fewer than 3 records were included and 0.458 when the criterion was applied to the same 80 hospitals. Both differ from 0.473, the eligible-stay-set estimate across all 193 eICU-CRD hospitals, and from the 0.354 reported in Table 3 for the restricted primary cohort. These figures answer different questions rather than estimating a single quantity: 0.510 and 0.458 compare eligibility definitions on a common 80-hospital denominator, while 0.473 and 0.354 are the eligible-stay-set and restricted-primary estimates of the hospital component itself. Table 3 retains the common 3-record cohort throughout to ensure comparable metrics, since the timing metrics cannot be computed without it. 0.510 is reported here as a sensitivity estimate and indicates the direction, not the magnitude, of the bias the criterion introduces. In MIMIC-IV, the criterion was immaterial, with between-unit η^2^ of 0.0268 without the 3-record minimum against 0.0271 with it. That a single institution loses 0.12% of stays to an availability criterion while a multisite database loses 10.22% is itself an expression of site-level heterogeneity in the record-generating process.

The excluded stays were characterized on every available attribute. In MIMIC-IV, the 84 excluded stays had a longer median ICU stay than those retained (median 6.53, IQR 3.42-13.11 vs median 2.41, IQR 1.57-4.48 days), with effectively identical standardized age (0.089 vs 0.119) and comorbidity count (–0.232 in both), so the criterion did not select younger or less comorbid patients. In eICU-CRD, the 13,583 excluded stays were closely comparable to those retained on median ICU length of stay (median 2.20, IQR 1.56-3.87 vs median 2.33, IQR 1.59-4.08 days) and median age (66 years in both), and had slightly lower ICU mortality (4.19% vs 5.33%). Exclusion was concentrated by site rather than by patient: the 10 hospitals with the highest exclusion rates supplied 26% (n=13,583) of all excluded stays, 6 of them excluding every stay under the 3-record minimum, with 4 further hospitals between 0.92 and 0.98. On the available characteristics, the criterion therefore removes stays that are not systematically sicker or otherwise distinct, and does so unevenly across contributing sites.

#### Variance Partition

A linear mixed model with random intercepts for hospital and unit within hospital, fitted to the unrestricted cohort of 96,518 stays across 68 hospitals, reproduced the hierarchy observed in η^2^. Hospital variance partition coefficients were 0.824 for the median charting interval, 0.671 for the fraction of the window inside gaps, 0.657 for the longest interval, 0.499 for gaps beyond 2 hours, and 0.434 for record count, vs 0.011-0.083 for unit within hospital. These are the unrestricted sensitivity estimates; the corresponding restricted values are those reported in Table 3. Because η^2^ and variance partition coefficients use different estimators and are computed here on different analysis sets, their numerical values are not expected to coincide; the concordant hierarchy indicates that the principal result does not depend on the sums-of-squares estimator.

Hospital attributes recorded in eICU-CRD. The database records 3 hospital attributes, collected by self-reported survey: bed capacity category, teaching status, and region. Among the 60 hospitals of the restricted primary cohort, teaching status was populated for all 60, region for 57, and bed capacity for 52. Marginal associations with the documentation metrics were weak: η^2^ was 0.013 to 0.021 for bed capacity, 0.000 to 0.012 for teaching status, and 0.010 to 0.103 for region, against a hospital component of 0.134 to 0.354 for the same metrics.

Conditioning each metric on all 3 attributes, among the 51 hospitals with every attribute populated, reduced the hospital component by 14.2% to 29.2%. Conditioning on 6 indicator coefficients removes part of the between-hospital structure by construction, so these shares were referred to a null obtained by permuting the attribute vectors across hospitals 10,000 times, holding each hospital’s stays and the joint distribution of the attributes fixed. Record count produced the largest excess over that null (29.2% observed against a null median of 9.6%; *P*=.006), which is nominally the smallest of the 10 tests but does not clear the Bonferroni-adjusted threshold of .005. The reductions for the longest interval and the median charting interval were nominally smaller still against their nulls (25.1% against 11.2%; *P*=.04; 23.1% against 11.1%; *P*=.06), and those for gaps beyond 2 hours and the fraction of the window inside gaps were indistinguishable from it (14.2% against 8.9%; *P*=.19; 15.0% against 8.7%; *P*=.15). In the unrestricted cohort the corresponding reductions were 5.7% to 12.5%, the largest excess again being for the longest interval (*P*=.009). No test in either cohort survives correction for the 10 tests performed.

The direction of association was consistent where present. Hospitals with 500 or more beds recorded a median of 29 (IQR 24-42) records per 24 hours at a 60-minute median interval, against 35 (IQR 24-58) records at 30 minutes in hospitals with 100 to 249 beds; teaching hospitals recorded a median of 29 (IQR 25-40) against 33 (IQR 24-53) for nonteaching hospitals. Region was the strongest single attribute for record count (η^2^ 0.103), with median counts of 38 (IQR 27-61) in the Midwest and 36 (IQR 27-56) in the West against 27 (IQR 22-37) in the South and 26 (IQR 24-30) in the Northeast. The remaining restricted-cohort tests gave *P* values from .04 to .19, and no association survived correction for the 10 tests performed.

Recorded structural attributes therefore produced the largest observed reduction in the site component for record volume, although it did not survive Bonferroni correction: the observed 29.2% reduction exceeded the 9.6% median reduction under the permutation null, but not the adjusted threshold of .005 for the 10 tests performed. No corresponding effect was detected for the interval or gap metrics. Most site-level variation remained unaccounted for by the 3 available structural attributes, which is consistent with the mechanisms named in the Discussion section and does not identify among them.

The 5 Gaussian mixed-model sensitivity fits on the unrestricted cohort converged under REML for 96,518 stays each, without warnings; the hospital-attribute analyses above are least-squares conditioning rather than mixed models.

### Global Threshold Consequence

Applied to the 88,560 eICU stays across the 60 hospitals of the restricted primary cohort, the pooled bottom-decile rule flagged 9006 (10.2%) stays, and the hospital-specific rules flagged 10,548 (11.9%), of which 4726 were common to both. Positive agreement was therefore 52.5% (4726/9006), and the Jaccard index was 0.319. In the unrestricted sensitivity analysis, applied to 96,518 stays across 68 hospitals, the pooled rule flagged 9906 (10.3%) stays and the hospital-specific rules 13,348 (13.8%), of which 5056 were common, giving 51.0% positive agreement and a Jaccard index of 0.278, with 13.6% (13,142/96,518) of all stays flagged by one rule but not the other. Positive agreement was 55.6% (3097/5570) at the 5th percentile and 63.7% (12,358/19,417) at the 20th. The within-site rule flagged more stays at every threshold (8.1%, 13.8%, and 25.0% against 5.8%, 10.3%, and 20.1%), a consequence of integer record counts and narrower within-site distributions that placed more stays at the cutoff value.

The disagreement is concentrated by site. In the restricted primary cohort, the share of a hospital’s stays flagged by the pooled rule had a median of 0.034, a 90th percentile of 0.315, and a maximum of 0.862: 15 of the 60 hospitals contributed less than 1% of their stays to the pooled bottom decile, and 6 hospitals accounted for half of all stays in it (Figure 5). In the unrestricted sensitivity analysis, the corresponding figures were a median of 0.010, a 90th percentile of 0.613, a maximum of 1.000, 34 of 68 hospitals below 1%, and 5 hospitals accounting for half, with 4 hospitals contributing every one of their stays; those 4 are among the 8 excluded as below the plausibility floor. Under a pooled threshold, therefore, the designation of low record density is carried principally by the hospital a patient was admitted to.

Within MIMIC-IV, the same procedure behaved differently. Across 9 care units, the 2 rules had 72.6% (7389/10,184) positive agreement at the decile (Jaccard 0.661), with 5.7% (3789/66,911) reclassified. The share of a unit’s stays flagged by the pooled rule ranged from 0.077 to 0.517, with no unit below 1%. This contrast indicates substantially greater threshold stability within a single institution than across multiple sites. The pooled decile cutoff was 10 heart rate records per 24 hours, well below the approximately hourly nurse-charting cadence observed in both cohorts. Four hospitals fell almost entirely below it. Whether those sites chart sparsely or contribute their nurse-charted data only partially cannot be determined from these data (see “Strengths and Limitations” section). Under either explanation, the pooled measure separates sites substantially rather than functioning primarily as a patient-level discriminator.

**Figure 5:**
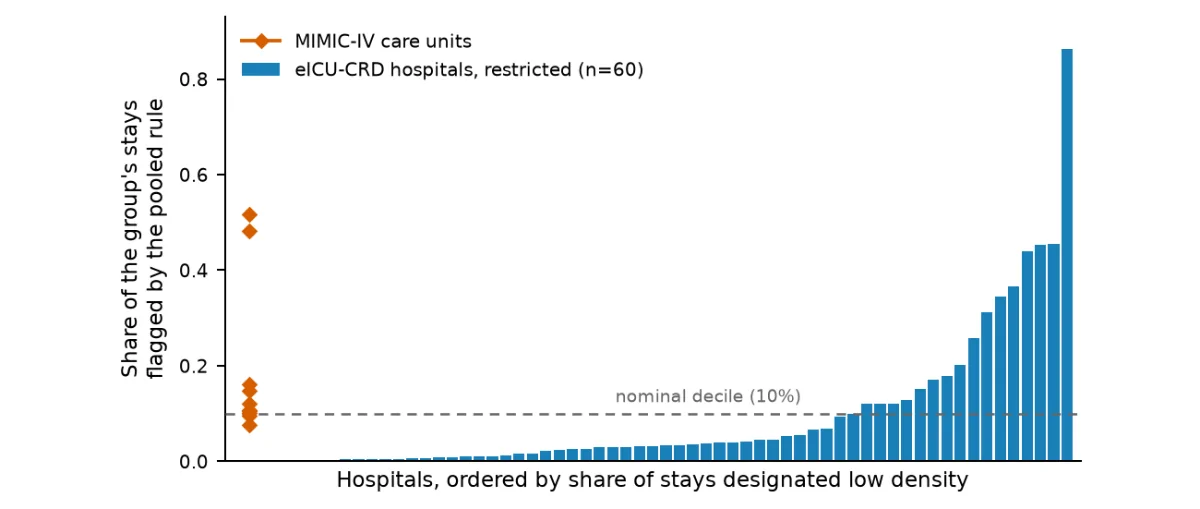
Share of adult intensive care unit stays at each hospital designated low density by the pooled bottom-decile heart-rate rule across the 60 eICU Collaborative Research Database (eICU-CRD) version 2.0 hospitals of the restricted primary cohort (nurse-charted stream, first 24 hours, US hospitals, 2014-2015); retrospective data quality study, with corresponding shares for the 9 Medical Information Mart for Intensive Care IV (MIMIC-IV) care units shown for contrast. Because record counts are discrete, the pooled and within-site percentile rules flag different proportions of stays (10.2% and 11.9% in the restricted cohort); the spread between groups shows how strongly the pooled designation is carried by site. Shares range from 0.000 to 0.862 across the 60 eICU-CRD hospitals of the restricted primary cohort, compared with 0.077 to 0.517 across MIMIC-IV care units.

### Replication in Oxygen Saturation and Respiratory Rate

The 4 replication analyses showed the same qualitative pattern across both additional variables (Table 5). Across both cohorts, nurse-charted cadence was 26-29 records per 24 hours with a 60-minute median interval for all 3 variables, vs 275-284 records with a 5-minute interval in the eICU-CRD monitor stream.

Bottom-decile agreement between the monitor and nurse streams for the same admissions was 0.137 for oxygen saturation and 0.125 for respiratory rate, against chance rates of 0.108 and 0.111, yielding ratios of 1.27 and 1.12. Heart rate yielded 1.01. On the plausibility-restricted cohort, the corresponding ratios were 1.02, 1.30, and 1.14 for heart rate, oxygen saturation, and respiratory rate, so the stream-selection finding is independent of the restriction. The 3 ratios are ordered by rank correlation between the streams (0.139, 0.072, and 0.024), so the 2 additional variables retain a modest shared component, whereas heart rate retains essentially none; in every case, agreement remains low despite both streams recording the same physiological variable over the same window.

The between-database η^2^ for record count was 0.861 for oxygen saturation and 0.895 for respiratory rate when MIMIC-IV was paired against the eICU monitor stream, and 0.020 and 0.017 when paired against the nurse stream, a reduction by factors of 43 and 53, respectively, compared with 37 for heart rate. Within the eICU nurse streams, the descriptive η^2^ pattern of a larger hospital than unit increment replicated for both variables; the replication covers that pattern rather than the order-free coefficients that carry the inferential statement. On the plausibility-restricted cohort for each variable, hospital explained 0.251 and 0.193 of the variance in median charting interval for oxygen saturation and respiratory rate and 0.322 and 0.333 of the variance in record count, against unit-within-hospital components of 0.041 to 0.050, closely reproducing the 0.251 and 0.354 obtained for heart rate on the same basis. Before restriction, estimated across all hospitals contributing eligible stays for each variable, the corresponding hospital values were 0.754 and 0.717 for median interval, against 0.005 and 0.010 for unit, and 0.465 and 0.453 for record count, against 0.034 for unit in both, reproducing the 0.761 against 0.003 and 0.473 against 0.029 obtained for heart rate on the same basis. Restricting each variable to hospitals with at least 500 stays moved these estimates by no more than 0.044. MIMIC-IV sat between the 29th and 46th percentiles of eICU units under the nurse pairing and at the 0th under the monitor pairing, for both variables.

The number of eICU hospitals contributing eligible stays varied by variable, with 193 for heart rate, 190 for respiratory rate, and 181 for oxygen saturation. The number meeting the 500-stay threshold also varied, with 68, 65, and 58, respectively. Nurse-stream coverage is therefore variable-specific, a further instance of the site-level heterogeneity documented here rather than a limitation peculiar to one analysis.

**Table 5:** Replication of the stream-concordance and variance findings across heart rate, oxygen saturation, and respiratory rate: retrospective data quality study of adult intensive care unit stays in the first 24 hours, Medical Information Mart for Intensive Care IV (MIMIC-IV) version 3.1 (1 US academic medical center, 2008-2022) and eICU Collaborative Research Database (eICU-CRD) version 2.0 (US hospitals, 2014-2015)^a^.

Quantity	Heart rate	Oxygen saturation	Respiratory rate	Note
MIMIC-IV median records/24 hours	26	26	26	Nurse stream
eICU-CRD nurse median records	29	28	27	—^b^
eICU-CRD monitor median records	284	275	279	—
Stream agreement, bottom decile	0.106	0.137	0.125	Observed
Chance under independence	0.105	0.108	0.111	Nurse marginal
Observed-to-chance ratio	1.01	1.27	1.12	—
Rank correlation between streams	0.024	0.139	0.072	Spearman ρ
Between-database η^2^ (count) vs monitor	0.960	0.861	0.895	—
Between-database η^2^ (count) vs nurse	0.026	0.020	0.017	Same MIMIC-IV data
Hospital η^2^ (record count)	0.354	0.322	0.333	eICU-CRD nurse stream
Unit within hospital (record count)	0.043	0.050	0.047	—
Hospital η^2^ (median interval)	0.251	0.251	0.193	eICU-CRD nurse stream
Unit within hospital (median interval)	0.031	0.041	0.048	—
MIMIC-IV percentile vs nurse stream	26th-41st	29th-43rd	30th-46th	Across MIMIC-IV units
MIMIC-IV percentile vs monitor stream	0th	0th	0th	—
eICU-CRD hospitals contributing	193	181	190	Nurse stream
Retained under the plausibility floor	60	50	56	—

^a^Oxygen saturation uses MIMIC-IV item ID 220277, with eICU-CRD nurseCharting “O_2_ Saturation” and vitalPeriodic “sao2”; respiratory rate uses item ID 220210 with “Respiratory Rate” and “respiration.” Alarm-limit, desaturation-limit, and ventilator-derived items are excluded throughout. Chance agreement is the nurse-stream marginal, which exceeds the nominal decile because integer counts produce ties at the cutoff. Variance components in this table are estimated on the plausibility-restricted cohort for each variable, with the floor of 12 observations per 24 hours applied using that variable’s own per-hospital median count, because a hospital may contribute one nurse-charted variable and not another. Applying the heart rate floor to all 3 variables instead gave nearly identical estimates, so the choice is not material. The heart rate column is computed on the same cohort as Table 3 and reproduces its values exactly. The restricted cohorts comprised 88,560, 77,967, and 82,486 stays for heart rate, oxygen saturation, and respiratory rate. The counts of hospitals contributing eligible stays before the floor were 193, 181, and 190, of which 68, 58, and 65 contributed at least 500 eligible stays. Restricting to hospitals with ≥500 stays changed them little: record-count hospital η^2^ was 0.441, 0.421, and 0.424, and median-interval hospital η^2^ was 0.763, 0.743, and 0.714, in heart rate, oxygen saturation, and respiratory rate order.

^b^Not available.

## Discussion

### Principal Findings

Contributing-site variation in vital-sign record density exceeded within-hospital unit variation for the 2 count outcomes, record count and the count of intervals exceeding 30 minutes, though by a smaller margin than an unrestricted analysis suggests; for the interval-structure metrics the 2 levels are not separable at this cohort size. Eight of 68 eICU-CRD hospitals recorded a median of fewer than 12 nurse-charted heart rate observations per stay, the 3 lowest recording a median of 3 (IQR 3-4), and per-hospital medians for heart rate and respiratory rate were highly correlated (Pearson *r*=0.973), though the variable-specific restriction retains different numbers of hospitals; a density that far below the conservative 2-hourly plausibility floor is more consistent with partial contribution of the nurse stream than with charting at that rate, although these data cannot exclude the latter. Excluding them reduced the hospital component for the median charting interval from 0.763 to 0.251 and for the fraction of the window inside gaps from 0.621 to 0.208, while record count fell only from 0.441 to 0.354. In the pooled restricted primary set, the database-first increment was 1.7%-8.1% of pooled variance, the subsequent hospital increment 10.0%-29.4%, and the unit-within-hospital increment 3.1%-5.1%. Intervals exceeding 30 minutes had a 23.1% database-first increment in that set and 24.9% in the pooled eligible-stay sensitivity set, because the metric counts hours in which an approximately hourly cadence was not met, a quantity that is related to record count by construction within a fixed window. Hours to first record showed a different pattern, with hospital and unit increments of 1.9% and 16.5%, respectively. Within a single institution, documentation volume and cadence were near-invariant across 9 named ICU types, whereas between sites on a common telehealth platform, median charting intervals differed by a factor of 4 after restriction and 12 before it.

We describe this as site-level rather than specifically institutional variation because the hospital component cannot be decomposed further with these data. It encompasses documentation practices, local system configurations, interface and integration arrangements, and the completeness of each site’s data contribution, and the present design does not separate them. Nor does a common data environment imply homogeneous bedside information systems across contributing hospitals: eICU distinguishes nurse flowsheet charting from monitored data at the level of the delivered dataset, not at the level of local architecture. The claim supported here concerns where the variance sits, not an ontological distinction between institution and architecture.

The practical consequence is clear: a pooled low-density threshold had 52.5% (4726/9006) positive agreement with site-specific thresholds in the restricted primary analysis, and 6 of 60 hospitals supplied half of what it flagged; before restriction the corresponding figures were 51% (5056/9906) and 5 of 68. A pooled rule intended to identify low-density stays largely identified hospitals. This was not merely an artifact of thresholding a discrete variable: the identical procedure across 9 care units within 1 institution produced 72.6% (7389/10,184) positive agreement vs 52.5% (4726/9006) across the 60 restricted hospitals and 51% (5056/9906) across all 68, and no unit contributed less than 1% of its stays, whereas 15 of 60 and 34 of 68 hospitals respectively did.

The pattern was not specific to heart rate. Oxygen saturation and respiratory rate reproduced all 4 replication analyses, including the collapse of the between-database η^2^ when streams were matched and the larger hospital than unit η^2^ increment. Within the scope of these 3 variables, the findings therefore characterize routinely nurse-charted vital-sign process measures rather than documentation of a single variable.

Three patterns that would ordinarily be read as findings were reproduced by measurement choices. The apparent architectural contrast between the 2 databases was produced by comparing a nurse-charted stream with a monitor stream; on equivalent streams, the between-database η^2^ fell from 0.960 to 0.026. The apparent cyclic temporal structure was produced by including stays that could not generate a full 24-hour window. The composition of the exposure itself included shift-anchored alarm-limit records that are not observations. Each is mundane on its own; together, they indicate that a plausible-looking EHR-derived measure can fail in several independent ways at once.

### Practice or Contribution

Site-level variation in a research database conflates documentation practice with the completeness of each site’s contribution, and this analysis separates them only partially. What it can do is bound the problem. The 8 excluded hospitals are separable on evidence rather than judgment, though the evidence is weaker than an absent contribution. Their median stay records 3 (IQR 3-4) to 9 (IQR 7-26) nurse-charted heart rate observations across 24 hours, against 32 for the cohort, so they do contribute to the stream but at a density below the conservative 2-hourly plausibility floor used in the primary analysis. The correlation of 0.973 between per-hospital heart rate and respiratory-rate medians shows that sparseness applies across variables rather than to one alone. Together with the weaker oxygen-saturation correlation of 0.610, this cross-variable concordance is consistent with a site-level contribution or completeness mechanism, but the pattern is not uniform and does not exclude variable-specific charting differences. The evidence for whole-stream contribution failure is therefore correspondingly qualified.

The restriction removes the most evident contribution-completeness outliers; it does not isolate documentation practice, and we do not claim that it does. A hospital component of 0.134 to 0.354 nonetheless remains among sites whose contribution is not implausible on these grounds, and it cannot be attributed to practice alone. Local system configuration, interface arrangements, and the completeness with which a site exports nurse-charted data all sit within it and are not distinguishable here. The claim supported here is that, within the multisite eICU-CRD nurse stream, variation in the 2 count outcomes resides more strongly at the contributing-site level than at the unit-within-hospital level, and that for the interval-structure metrics the 2 levels cannot be separated at this cohort size. The cross-database pooled increments are descriptive because they depend on factor-entry order. Identifying which site-level mechanism is responsible would require information on local documentation systems that neither database provides.

Three specific mechanisms deserve naming. The eICU-CRD hospitals share a telehealth platform, and remote monitoring may itself alter local documentation: where a remote team reviews patients continuously, bedside nurses may chart less often, because remote review substitutes for some local surveillance, or more often, because remote review generates queries that prompt documentation. MIMIC-IV has no equivalent arrangement, so the cross-database contrast includes a workflow difference that neither database records and whose direction cannot be determined here.

The contributing hospitals also do not share a bedside EHR. Local EHR configurations, including default flowsheet cadences, could produce different record densities without any difference in nursing practice: a default 2-hourly template and a default hourly one would not yield the same count. Vendor identity is not recorded in eICU-CRD, so this cannot be tested; it is among the more plausible mechanisms inside the hospital component, given that the component survives conditioning on record volume.

A third mechanism is organizational rather than technical. Sites differ in data-quality maturity: in whether documentation completeness is monitored, whether staff are aware that flowsheet entries are reused for research and quality measurement, and whether export pipelines are revalidated after configuration changes. If maturity differences contribute materially to the site component, the implied interventions are organizational, namely completeness monitoring, feedback to charting staff, and validation of research extracts after system changes, rather than statistical adjustment at analysis time. These data cannot apportion the component across mechanisms, but the distinction determines what a receiving institution should do about it.

### Comparison With Prior Work

That documentation practice influences EHR-derived measures is established. Informative presence and informative observation describe dependence of encounter timing and measurement decisions on latent health state, and methods exist to accommodate both [4,5]. Workflow and documentation habits can directly affect EHR-derived quality measures [3]. Our contribution is not to show that such dependence exists, but to show that within the multisite eICU-CRD data, more of the variation in the 2 count outcomes resides at the contributing-site level than at the unit-within-hospital level, while for the interval-structure metrics the 2 levels are not separable at this cohort size. This localization is actionable because it identifies the level to be modeled while leaving open which site-level mechanisms—practice, configuration, integration, or completeness—are responsible in a given dataset.

The cross-site differential in eligibility is another instance of the same phenomenon. An availability criterion that removes roughly one stay in a thousand at a single institution removes 1 in 10 across a multisite database and does so unevenly across sites. Any study applying a minimum-records rule to multisite EHR data therefore selects on site as well as on patient.

Three features distinguish the present analysis from the nearest published work. Data-quality effects on integration pipelines have been demonstrated by injecting structured noise into MIMIC-III and observing the consequences [15]; here, degradation is observed rather than simulated, and its magnitude is estimated across 194 sites. Multisource coherence has been quantified across 4 countries in a cohort of 652 participants [14], and completeness has been comprehensively assessed within 1 institution [13]; the present design combines multisite scale with within-institution replication so that between-site and within-site variation are estimated using the same metrics. The decomposition is also accompanied by clustered interval estimates and a second variance estimator.

The stream-selection result illustrates an additional failure mode. When a database contains multiple tables that measure the same physiological variable, source-table choice can materially affect which patient stays are identified as having low record density. For heart rate, bottom-decile agreement matched the independence expectation; oxygen saturation and respiratory rate remained only modestly above it. This is not merely a difference in scale or cadence that normalization can remove: the streams select substantially different populations.

### Implications

Three practical requirements follow. First, the generating stream must be specified: a study reporting vital-sign record density should state the source table and item identifiers, and if a database offers several, justify the choice. Second, the analytic window must be enforced by restriction rather than assumed, since stays ending before the window close carry a mechanical density deficit that is correlated with admission time. Third, site-level variation must be modeled explicitly in any cross-site comparison, because it is the largest identified component for the count outcomes, is not separable from unit-level variation for the interval metrics, and is not removed by describing the recording architecture. Where the purpose is to identify relative low density within a site, thresholds should be calibrated within site; pooled thresholds should not be assumed transportable across sites without adjustment or external validation. We do not claim the within-site rule is the correct one—a within-site percentile rule assigns low-density status relative to each hospital by construction and would therefore erase genuine between-site differences—only that the 2 rules answer different questions, and that the pooled rule answers a question about sites.

A statistically low value is also not necessarily a clinical deficit. A stay in the pooled bottom decile at a cutoff of 10 records per 24 hours may still satisfy a protocol appropriate to a stable patient, and a stay above the cutoff may fall short of the protocol that applies to it. The designation identifies relative position in a distribution, not adequacy against a standard of care, and the two should not be conflated when such measures are used for monitoring. For the specific proposal that low record density indicates care discontinuity or reduced recording intensity, our results counsel caution. Within a site, the measure was stable enough that local monitoring may be meaningful; between sites, it is not comparable without adjustments that these data cannot provide.

### Why No Outcome Association Is Interpreted

Record density is charted by clinicians in response to patient state and is therefore partly a measure of acuity. Separating documentation behavior from acuity requires a severity instrument that does not itself derive from the same charting process, and no such instrument could be harmonized across these cohorts: stream-independent components were recorded too sparsely, whereas adequately recorded components arose from the same nurse-charting process. Density-mortality estimates shifted substantially, even reversing direction, in eICU-CRD with the adjustment instrument. Mortality models are reported in the Severity Harmonization section only to document this instability; promoting any one specification as a finding would misrepresent an analytic choice as an empirical result. The variance decomposition explains why: an exposure whose dominant variance component is the contributing site is entangled with site-level data-generating processes before patient-level adjustment begins.

### Strengths and Limitations

Strengths include 2 large cohorts with complementary designs, allowing within- and between-institution variation to be estimated separately; clustered bootstrap inference for the principal decomposition; verification of exposure composition against the source item dictionary; and a principal analysis that does not depend on an outcome model and therefore does not require resolution of the acuity-adjustment problem that prevents interpretation of the mortality models.

Several mechanisms that could generate site-level variation cannot be examined in these resources because neither database records them. eICU-CRD does not report bedside EHR vendor, nurse-to-patient ratio, or the local configuration of remote monitoring; neither database records the curation decisions applied upstream of release. It does record teaching status, bed-capacity category, and region, which we examined directly; the largest reduction they produced, in record volume, did not survive correction for the 10 tests performed, and no reduction was detected elsewhere (see “Sensitivity Analyses” section). The limitations set out below therefore identify what the site component may contain rather than testing between the candidates, and closing them would require site-level metadata that research databases of this kind do not currently carry. That requirement is itself a finding: a resource intended to support cross-site evaluation of documentation-derived measures would need to release the operational attributes that plausibly generate the site component, not only the clinical records it conditions.

Limitations include incomplete comparability of site coverage across streams. eICU-CRD nurse-charted heart rate met the 500-stay threshold at 68 hospitals vs 79 for the monitor stream; of the 11 hospitals affected, 1 contributed no nurse-charted heart rate, and the remainder contributed 13%-80% of their monitor-stream volume. Per-hospital median counts as low as 3 records per 24 hours are far below the approximately hourly cadence observed in both cohorts and are more consistent with partial data contribution than with the predominant charting pattern; the data cannot fully separate documentation practice from data completeness at the site level, so the hospital component should be read as encompassing both. MIMIC-IV contributes a single institution, so neither the between-database η^2^ nor the database-first increment can be separated from institution-specific differences on the MIMIC-IV side, and neither should be interpreted as a pure database effect. The analytic unit also differs between the cohorts: MIMIC-IV is restricted to the first ICU stay of each hospital admission, whereas eICU-CRD retains repeat unit admissions within a hospitalization, so a patient readmitted to an eICU-CRD unit contributes more than once, which may introduce within-patient dependence not represented by the hospital- and unit-level random effects. Replication covered 3 vital signs charted at a common nurse cadence; measures charted on other schedules, such as laboratory or ventilator-derived variables, were not examined and may behave differently. Both cohorts are US-based; eICU-CRD reflects 2014-2015, whereas MIMIC-IV version 3.1 spans 2008-2022. A third public ICU database, SICdb, could not extend the nurse-vs-monitor comparison because its data model does not provide a directly analogous pair of nurse-charted and monitor tables [22].

Charting practice is not static across that interval. Alarm-management policy in particular changed materially over the period, and institutional responses to alarm burden may alter both the volume and the anchoring of nurse-charted observations. Because the database-first increment contrasts a single institution observed over 15 years against a multisite collection observed over two, era and site are not separable here, and the database-first increment should not be read as a contemporaneous comparison.

Documentation practice is also shaped by nurse staffing models, accreditation requirements, and medico-legal expectations that differ substantially between health systems, and the levels at which variance sits may differ correspondingly. These estimates should not be assumed to transfer to datasets governed by other documentation regimes. The design principles drawn from them, namely specifying the generating stream, enforcing the analytic window, and modeling site explicitly, are more transferable than the magnitudes.

Restricting timing metrics to stays completing 24 hours is necessary because a truncated stay cannot generate a complete interval series, but it removes patients who died or were discharged within the first day. Those stays are plausibly both the most intensively monitored and the most irregularly documented, so their removal may smooth the charting variance estimated here; the direction of the resulting bias on the variance components cannot be determined from these data. The count end point was additionally computed over all stays completing the window with zero assigned where no record was present, and that specification widened rather than narrowed the between-hospital component.

Both cohorts comprise ICU admissions, so the unit-within-hospital component is estimated only across ICU subtypes, which share a broadly similar intensive-care monitoring context; nurse-to-patient ratio is not recorded in either database and is not among the attributes compared. A second constraint on the unit level is that 26 of the 60 hospitals in the restricted primary cohort contribute a single unit type, as do 30 of 68 unrestricted and 151 of 193 contributing eligible stays. In a single-unit-type hospital, the unit variance cannot be separated from the hospital component, so the hospital coefficients reported here are likely upper bounds and the unit coefficients likely lower bounds. The finding that the unit component is the smaller of the two for the count outcomes is therefore specific to that setting. Vital-sign recording frequency is protocol-driven by clinical indication, and a decomposition spanning genuinely different care settings would be expected to place substantially more variance at the unit level. The conclusion supported here is that within intensive care the volume of recording is site-dependent rather than unit-dependent, while for the interval and gap structure the 2 levels are not separable at this cohort size and the unit coefficient is the larger point estimate for the count of gaps beyond 2 hours; it is not a general claim that care setting is unimportant to documentation density.

MIMIC-IV and eICU-CRD are curated research products rather than raw exports. Each has passed through extraction, mapping, deidentification, and versioning steps whose choices are not visible downstream. The alarm-limit finding reported here is itself an instance: an item-composition decision made upstream propagated into an exposure definition. We have not established that item mappings are applied identically across contributing eICU-CRD hospitals, that deidentification preserved temporal structure uniformly, or that MIMIC-IV version 3.1 is comparable in this respect to earlier versions. The hospital component may therefore include curation heterogeneity alongside practice, configuration, and export completeness.

Two features of the estimation were examined directly rather than assumed (see “Sensitivity Analyses” section). Requiring 3 records conditions inclusion on the process under study; admitting stays below that threshold increased rather than decreased the between-hospital component for record count, so the criterion was conservative here, although it removes 10.22% (13,583/132,900) of eICU-CRD stays and that exclusion is unevenly distributed across sites. The mixed-model variance partition confirmed a substantial hospital component and reproduced the site-dominant hierarchy in the unrestricted sensitivity analysis. In the restricted cohort, hospital and unit components were closer for some interval metrics, so the magnitude of the separation between the 2 levels depends on both the metric and the estimator, even though its direction does not for the 2 count outcomes that carry the principal claim. Those 2 outcomes are also not independent, since within a fixed window the count of intervals exceeding 30 minutes is inversely related to record count by construction (Pearson *r*=–0.615 restricted, –0.376 unrestricted), so their agreement is a consistency check on one measure of charting density rather than confirmation across 2 separate outcomes. Neither analysis addresses the more fundamental limitation that the hospital component conflates documentation practice with data completeness.

### Conclusions

In these 2 databases, site-level variation in vital-sign record density exceeded within-hospital unit variation for the 2 count outcomes, record count and the count of intervals exceeding 30 minutes, under the Gaussian and the negative binomial specifications alike, the hospital and unit intervals separating in every case (Table 3). For the interval-structure metrics, the 2 components are not separable at this cohort size, and for the count of gaps exceeding 2 hours the unit coefficient is the larger on the observed scale (0.308 vs 0.280). We therefore restrict the claim to these 2 count outcomes, and note that unit-level variation is not negligible but concentrated in the timing and gap structure of recording rather than in the counts. Apparent cross-database differences were reproduced by source-table choice, and an apparent temporal association was largely explained by analytic-window truncation. Comparative use of this and similar EHR-derived process measures requires specifying the generating stream, enforcing the analytic window, and explicitly modeling site-level variation. The site component observed here encompasses both documentation practice and data completeness; separating these mechanisms will require data on local system configuration that these cohorts do not provide.

## Appendix

Multimedia Appendix 1 Data dictionary and stream provenance.

## Abbreviations

APACHE: Acute Physiology and Chronic Health Evaluation
EHR: electronic health record
eICU-CRD: eICU Collaborative Research Database
HIPAA: Health Insurance Portability and Accountability Act
ICU: intensive care unit
MIMIC-III: Medical Information Mart for Intensive Care III
MIMIC-IV: Medical Information Mart for Intensive Care IV
OR: odds ratio
REML: restricted maximum likelihood
STROBE: Strengthening the Reporting of Observational Studies in Epidemiology

## Data Availability

Medical Information Mart for Intensive Care IV version 3.1 [18] and eICU Collaborative Research Database version 2.0 [19] are available to credentialed users through PhysioNet under their respective data use agreements and cannot be redistributed by the author. The analysis code implements the vital-sign exposure definitions and source-stream extraction, 24-hour record density and interval metrics, nurse-vs-monitor stream comparisons, database-, hospital-, and unit-level variance decompositions, hospital-clustered bootstrap inference, mixed-effects variance-partition sensitivity analyses, pooled vs site-specific threshold analyses, admission-hour models, and replication analyses for heart rate, oxygen saturation, and respiratory rate. The final code and aggregate analysis outputs are archived in a versioned public repository [23]. Multimedia Appendix 1 gives the stream provenance, source-field selectors, record and metric definitions, and eligibility rules needed to reconstruct the analysis from the source databases. No patient-level data are redistributed.
