# Supplementary material for: Data Stream and Hospital-Level Variation in Intensive Care Unit Vital-Sign Record Density Across Medical Information Mart for Intensive Care IV and eICU Collaborative Research Database: Retrospective Data Quality Study
Source: JMIR Med Inform. 2026 Oct 29;14:e99638. doi: 10.2196/99638 (PMC13760329; doi:10.2196/99638)
Supplement: Supplementary file 1 [file medinform_v14i1e99638_app1.docx]

Multimedia Appendix 1. Data dictionary and stream provenance

*Companion to: Data-Stream and Hospital-Level Variation in ICU Vital-Sign Record Density Across MIMIC-IV and eICU-CRD: Retrospective Data Quality Study. This appendix defines the operational provenance of every data stream used, so that the analysis can be reproduced from the source databases without inspecting the analysis code.*

# A1. Stream provenance

| **Attribute** | **MIMIC-IV chartevents** | **eICU-CRD nurseCharting** | **eICU-CRD vitalPeriodic** |
| --- | --- | --- | --- |
| Generating process | Charted ICU flowsheet observation | Nursing-charting observation; entered directly or interfaced from the bedside electronic medical record | Continuously interfaced bedside-monitor data archived as 5-minute medians |
| Heart rate selector | chartevents itemid 220045 | nursingchartcelltypevallabel or nursingchartcelltypevalname containing 'heart rate', case-insensitive; value read from nursingchartvalue | column heartrate |
| Oxygen saturation selector | chartevents itemid 220277 | nursingchartcelltypevalname = 'O2 Saturation' | column sao2 |
| Respiratory rate selector | chartevents itemid 220210 | nursingchartcelltypevalname = 'Respiratory Rate' | column respiration |
| Excluded items | HR alarm limits 220046, 220047; SpO2 alarm and desaturation limits 223769, 223770, 226253; RR alarm limits 224161, 224162; ventilator-derived RR 224688, 224689, 224690 | none (no alarm-limit rows carry these value names) | none |
| Timestamp field | charttime, converted to minutes from ICU admission | nursingchartoffset, minutes from unit admission | observationoffset, minutes from unit admission |
| Observed cadence, first 24 h | mean 28.0 records, median 26, median interval 60 min | mean 34.9 records, median 29, median interval 60 min | mean 273.5 records, median 284, median interval 5 min |
| Plausible value range | Heart rate 20-250 bpm; oxygen saturation 50-100%; respiratory rate 4-60 breaths/min | Same ranges as MIMIC-IV | Same ranges as MIMIC-IV |
| Sites | One US academic medical center | US hospitals on a shared telehealth platform | Same eICU-CRD hospital network; variable- and stream-specific coverage differs |
| Period | 2008-2022 | 2014-2015 | 2014-2015 |

# A2. What constitutes one record

A record is a single timestamped row in the source table carrying a numeric value within the plausible physiological range, with its timestamp falling inside the analytic window. It is not a measurement event, a distinct time-value pair, or an archived monitor value: a row is counted once, whatever else shares its timestamp.

Rows with non-numeric or missing values were dropped before counting. Rows were retained where the value fell within the variable-specific plausible range: 20 to 250 beats per minute for heart rate, 50% to 100% for oxygen saturation, and 4 to 60 breaths per minute for respiratory rate. The same bounds were applied in both databases and in both eICU-CRD streams.

The analytic window is the first 24 hours from ICU admission (MIMIC-IV) or unit admission (eICU-CRD): offsets of at least 0 and fewer than 1440 minutes.

eICU-CRD offsets are recorded relative to unit admission rather than wall-clock time. Two consequences follow. The on-the-hour charting fraction, which requires the clock hour of each record, is computed for MIMIC-IV only. An approximate eICU-CRD analogue could be constructed by adding each record offset to the unit admission clock time in patient.unitadmittime24, the field the admission-hour analysis uses. The derivation was not part of the analysis plan; a site-level on-the-hour fraction obtained this way would nevertheless bear on the default flowsheet cadence discussed in the manuscript. The admission-hour analysis, which requires only the clock hour of admission rather than of each record, is computed for eICU-CRD from patient.unitadmittime24.

# A3. Duplicate and simultaneous timestamps

Rows sharing an identical timestamp for the same stay and variable were NOT collapsed. Each is counted as a separate record, and each contributes a zero-length interval to the interval statistics. No de-duplication, rounding, or time-binning was applied at any stage.

This is a deliberate choice rather than an oversight: collapsing simultaneous entries would impose a definition of a measurement event that the source tables do not support uniformly across sites, and would itself introduce a site-dependent transformation into a study about site dependence.

In these data the choice makes no difference. Among 2,444,918 MIMIC-IV heart-rate records no timestamp was duplicated within a stay. In eICU-CRD, 2 of 5,223,657 nurse-charted records and 17 of 45,164,349 monitor records shared a timestamp with another record for the same stay and variable. Zero-length intervals accounted for under 0.001% of intervals in every stream, and no reported metric changed when identical timestamps were collapsed. Counting rows and counting distinct timestamps are therefore equivalent operations here.

# A4. Derived metrics

All metrics are computed per stay from the ordered sequence of in-window record offsets. Intervals are the successive differences of that sequence, so a stay with n records yields n-1 intervals. The interval metrics therefore exclude the time from ICU or unit admission to the first record and the time from the last record to the close of the window: neither edge is counted toward the longest interval or the fraction of the window inside gaps. Where the first record is delayed the omission is material, as in the MIMIC-IV cardiac vascular ICU, whose median time to first record is 2.57 hours.

Record count: the number of in-window records. Hours to first record: the smallest offset, in hours. Median interval, interval interquartile range, and longest interval: the median, the 75th minus the 25th percentile, and the maximum of the interval series, in minutes. Gaps >30 min and gaps >2 h: the counts of intervals strictly exceeding 30 and 120 minutes. Fraction of window in gaps: the summed length of intervals exceeding 120 minutes, divided by 1440. On-the-hour fraction (MIMIC-IV only): the share of records charted within the first five minutes of a clock hour.

# A5. Primary cohort eligibility and grouping

A stay is eligible where it completes the 24-hour window (MIMIC-IV ICU length of stay of at least one day; eICU-CRD unit discharge offset of at least 1440 minutes) and carries at least three in-window records of the variable concerned. The three-record minimum is applied within each source stream separately, so stream-specific cohorts differ.

Variance in the pooled decomposition is reported as nested increments under a stated factor ordering rather than as components, because the three factors are perfectly nested and the sequential values depend on entry order; the manuscript reports the database-first increment, the subsequent hospital increment and the unit-within-hospital increment, and the order-free variance partition coefficients alongside them.

Alternative analysis sets are used where a question requires them and are specified in the manuscript: clustered inference on the eligible-stay set resamples all 193 eICU-CRD hospitals contributing eligible stays, mixed models and per-hospital summaries use the 68 hospitals contributing at least 500 eligible stays, and the admission-hour models are reported both for all stays and for stays completing the 24-hour window.

Hospitals are grouped by eICU-CRD hospitalid; MIMIC-IV is treated as a single hospital. Care units are grouped by the hospital identifier concatenated with unittype in eICU-CRD and by careunit in MIMIC-IV, so unit is nested within hospital by construction. Hospital-level summaries use hospitals contributing at least 500 eligible stays; the plausibility-restricted primary analysis additionally requires a per-hospital median of at least 12 eligible records per 24 hours.

# A6. Analysis sets

Every analysis set used in the manuscript is listed below with the rule that defines it, so that each carries one label throughout. Counts are stays, with the site count given where it applies. The restricted primary cohort carries the principal estimates; the unrestricted cohort differs from it only by the plausibility floor, and the remaining sets are used where a specific question requires them.

| **Analysis set** | **Definition** | **Stays** | **Sites** |
| --- | --- | --- | --- |
| MIMIC-IV frame | icustays table, MIMIC-IV v3.1 | 94,458 | 1 center |
| MIMIC-IV cohort | first ICU stay of each hospital admission, with valid admission and discharge timestamps | 85,228 | 1 center |
| MIMIC-IV admission-hour set | cohort stays carrying a heart-rate count and an admission hour; no window restriction | 85,133 | 1 center |
| MIMIC-IV completed-window set | ICU length of stay ≥1 day | 67,224 | 1 center |
| MIMIC-IV analytic set | completed-window stays with ≥3 in-window heart-rate records | 67,140 | 1 center |
| eICU-CRD frame | patient table, eICU-CRD v2.0 | 200,859 | 208 hospitals |
| eICU-CRD admission-hour set | unit stays present in both source streams; 177,176 after listwise deletion on age and sex; no window restriction | 177,198 | — |
| eICU-CRD admission-hour peak set | the 177,176 admission-hour set stays remaining after listwise deletion that complete the 24-hour window, at hospitals contributing ≥500 such stays, stays with no nurse-charted record retained as zero; used for the per-hospital peak-time summary only | 99,047 | 71 hospitals |
| eICU-CRD completed-window set | unit discharge offset ≥1440 min | 132,900 | — |
| eICU-CRD eligibility-sensitivity set | completed-window stays at hospitals contributing ≥500 such stays, stays with no nurse-charted record retained as zero | 108,305 | 80 hospitals |
| eICU-CRD eligible-stay set | completed-window stays with ≥3 in-window nurse-charted records | 119,317 | 193 hospitals |
| eICU-CRD unrestricted cohort | eligible stays at hospitals contributing ≥500 of them | 96,518 | 68 hospitals |
| eICU-CRD restricted primary cohort | unrestricted cohort further restricted to hospitals whose median eligible stay records ≥12 observations per 24 h | 88,560 | 60 hospitals |
| eICU-CRD monitor-stream set | completed-window stays with ≥3 in-window vitalPeriodic records | 130,294 | — |
| eICU-CRD paired-stream set | eligible in both source streams | 117,572 | — |
| Complete-attribute unrestricted set | unrestricted cohort hospitals with all three recorded attributes populated | 86,395 | 59 hospitals |
| Complete-attribute restricted set | restricted primary cohort hospitals with all three recorded attributes populated | 78,437 | 51 hospitals |
| Pooled restricted primary set | MIMIC-IV analytic set plus the restricted primary cohort | 155,700 | 61 sites |
| Pooled eligible-stay sensitivity set | MIMIC-IV analytic set plus the eligible-stay set; 282 unit cells | 186,457 | 194 sites |

The MIMIC-IV and eICU-CRD admission-hour model sets do not pass through the 24-hour-window restriction and are therefore parallel to the completed-window pathway rather than stages of it; the participant flow diagram shows them as separate branches. The eICU-CRD admission-hour peak set is the one exception: it is a completed-window subset of the eICU-CRD admission-hour set, used only for the hospital-level peak-time summary.

# A7. Reproduction

MIMIC-IV version 3.1 and eICU-CRD version 2.0 are available to credentialed users through PhysioNet under their respective data use agreements and cannot be redistributed. The analysis code and every aggregate output underlying the manuscript are archived as version 1.1.4 at doi:10.5281/zenodo.23540691, with the working repository at github.com/yngvemikkelsen/icu-record-density-transportability. The archive includes the plausibility-restriction and threshold-sweep analyses, the fitted negative binomial and zero-truncated negative binomial generalized linear mixed models, the hurdle specification for gaps exceeding 2 hours, the bias-corrected bootstrap and profile-likelihood interval routines, the hospital-attribute conditioning and its 10,000-permutation null, the duplicate-timestamp and covariate-missingness checks, the APACHE-availability sensitivity analysis, and the entry-order sensitivity analysis, together with a script that runs the whole pipeline in dependency order. No patient-level data are redistributed.
